# Photo-driven redox-neutral decarboxylative carbon-hydrogen trifluoromethylation of (hetero)arenes with trifluoroacetic acid

**DOI:** 10.1038/ncomms14353

**Published:** 2017-02-06

**Authors:** Jin Lin, Zhi Li, Jian Kan, Shijun Huang, Weiping Su, Yadong Li

**Affiliations:** 1 State Key Laboratory of Structural Chemistry, Fujian Institute of Research on the Structure of Matter, Chinese Academy of Sciences, Fujian 350002, China; 2 Department of Chemistry, Tsinghua University, Beijing 100084, China

## Abstract

Catalytic oxidative C–H bond functionalization reactions that proceed without requiring stoichiometric amounts of external oxidants or pre-functionalized oxidizing reagents could maximize the atom- and step-economy in chemical syntheses. However, such a transformation remains elusive. Here, we report that a photo-driven catalytic process enables decarboxylative C–H trifluoromethylation of (hetero)arenes with trifluoroacetic acid as a trifluoromethyl source in good yields in the presence of an external oxidant in far lower than stoichiometric amounts (for example, 0.2 equivalents of Na_2_S_2_O_8_) using Rh-modified TiO_2_ nanoparticles as a photocatalyst, in which H_2_ release is an important driving force for the reaction. Our findings not only provide an approach to accessing valuable decarboxylative C–H trifluoromethylations via activation of abundant but inert trifluoroacetic acid towards oxidative decarboxylation and trifluoromethyl radical formation, but also demonstrate that a photo-driven catalytic process is a promising way to achieve external oxidant-free C–H functionalization reactions.

## Abstract (web summary)

C–H bond functionalization has become an important tool for many organic transformations, however such reactions typically require stoichiometric amounts of external oxidants. Here the authors report the photo-driven decarboxylative C–H trifluoromethylation of arenes using only low amounts of oxidant.

C–H bond functionalization, due to its simplified synthetic routes and readily available starting materials, provides a potentially atom- and step-economic synthetic approach complementary to the conventional functionality-based synthetic methods^1^,^2^,^3^,^4^,^5^. The potential power of the C–H functionalization strategy in organic synthesis has been clearly demonstrated by its application to natural product synthesis^6^, medicine synthesis^7^,^8^, late-stage functionalization of complex molecules^9^,^10^ and material preparations^11^. Full realization of the intrinsic advantage of the atom- and step-economy of C–H functionalization reactions, however, remains a significant challenge because such reactions generally require either stoichiometric amounts of external oxidants (Fig. 1a) such as metal salt oxidants or oxidizing reactants that are often prepared via multistep procedures (Fig. 1b). Natural photosynthesis, a process that enables photo-driven water splitting to molecular dioxygen and atomic hydrogen via coupling of the water oxidation half-reaction with the proton reduction half-reaction^12^, offers a blueprint for the design of oxidative C–H functionalization reactions that proceed without reliance on any external oxidant or oxidizing reactant.

On the other hand, the importance of trifluoromethyl (CF_3_) group-containing compounds in pharmaceuticals, agrochemicals and materials has spurred tremendous efforts devoted to the development of methods for the incorporation of trifluoromethyl group into molecular frameworks and the discoveries of trifluoromethylating reagents^13^. As a result of the inventions of a diverse range of trifluoromethylating reagents including nucleophilic ones (CF_3_^−^) and electrophilic ones (CF_3_^+^), a variety of metal-catalysed direct C–H trifluoromethylations have been achieved^14^,^15^,^16^,^17^,^18^. Benefiting from the identification of various precursors that are capable of generating trifluoromethyl radicals through regular oxidation^19^ or photoredox catalysis^20^ under mild conditions, the radical-based reactions have evolved into an efficient approach to construct C-CF_3_ bonds. In spite of these remarkable advances, the commonly used trifluoromethylating reagents are very expensive due to their time-consuming and multistep syntheses, which prompt chemists to develop readily available, cost-effective surrogates of existing trifluoromethylating reagents.

Herein, we report a photo-driven decarboxylative C–H trifluoromethylation of nonactivated (hetero)arenes with readily available, low-cost trifluoroacetic acid as a trifluoromethylating reagent. Using Rh-modified TiO_2_ NPs as a catalyst, our reaction can occur in the absence of external oxidant to afford a trifluoromethylation product in a modest yield with H_2_ release as a driving force for the reaction. Our findings demonstrate that the underlying redox chemistry of natural photosynthesis can operate in C–H functionalization reactions, which may provide an opportunity to maximize the step- and atom-economy of such reactions.

## Results

### Reaction design

Minisci reactions enable the use of alkyl carboxylic acids for C–H alkylation of electron-deficient pyridines via oxidative decarboxylation of alkyl carboxylic acids^21^. Recently, Minisci reaction of aryl carboxylic acids has been realized by us^22^. Minisci reactions have implied that trifluoroacetic acid (TFA) may act as a trifluoromethylating reagent via oxidative decarboxylation to generate trifluoromethyl radical^23^,^24^,^25^. In terms of its stability, ready availability and low price, trifluoroacetic acid is an intrinsically advantageous trifluoromethylating reagent. The extraordinary inertness of trifluoroacetic acid (electro-oxidation of TFA anion to generate trifluoromethyl radical occurs at potentials higher than +2.24 V versus the saturated calomel electrode (SCE)^26^), however, largely retards its application to the trifloromethylation reactions. For example, the decarboxylative C–H trifluoromethylation mediated by a stoichiometric amount silver salt required 2 equivalents of K_2_S_2_O_8_ as oxidant and tolerated only a few electron-deficient substituted benzenes^25^.

A recent report on using trifluoroacetic anhydride, a derivative of trifluoroacetic acid, for the C–H trifluoromethylation provides an alternative solution for the inexpensive trifluoromethyl source^27^, which, however, requires the use of stoichiometric pyridine *N*-oxide as a sacrificial oxidant.

**Figure 1:**
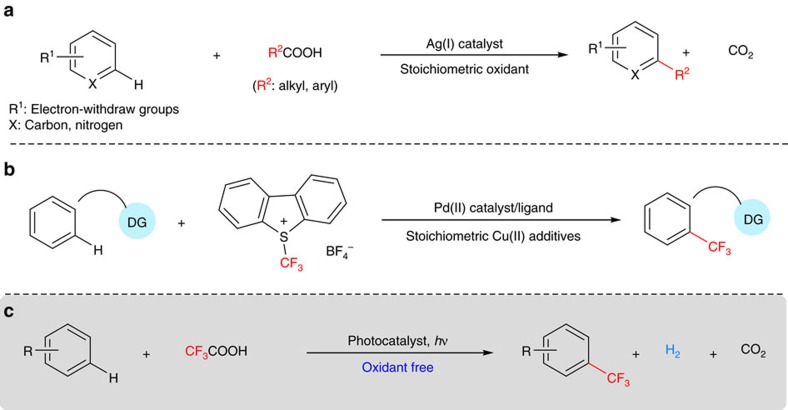
Catalytic oxidative C–H functionalization. (**a**) Minisci reaction: the example of the C–H functionalization with stoichiometric external oxidant. (**b**) C–H trifluoromethylation with Umemoto reagent: the example of C–H functionalization with pre-synthesized oxidazing reagent. (**c**) Photo-driven C–H trifluoromethylation without the reliance on external oxidant (this work).

TiO_2_-based NPs are extensively used as photocatalysts for the photo-driven water splitting processes^28^. Significant redox potential (+3.1 V versus SCE)^29^ of the photo-generated hole in the valence band of TiO_2_, in principle, would make it capable of oxidizing trifluoroacetic acid to generate trifluoromethyl radicals via decarboxylation and therefore trigger the trifluoromethylation reaction. Accordingly, we envisioned that TiO_2_-based NPs could catalyse the photo-driven decarboxylative C–H trifluoromethylation reaction with trifluoroacetic acid as a trifluoromethyl source (Fig. 1c). More importantly, the similarity to the photocatalytic water splitting process also implies the possibility that this decarboxylative C–H trifluoromethylation reaction proceeds without the need for the stoichiometric amount of external oxidants. Only one example of TiO_2_-promoted photo-driven decarboxylative C–H trifluoromethylation reaction of benzenes in trifluoroacetic acid that required stoichiometric amount of silver trifluoroacetate as an oxidant and gave poor yields indicates that there is significant room to improve the photocatalyst^23^.

### Establishment of photo-driven trifluoromethylation

We started our investigations by testing the trifluoromethylation of benzene with trifluoroacetic acid as a trifluoromethyl source. Numerous trials were performed to screen the reaction parameters such as different solvents, TiO_2_-based photocatalysts, light sources and external oxidants (details are provided in Supplementary Table 1). Selected results are presented in Table 1. Most strikingly, in this process C–H trifluoromethylation can happen with no sacrificial oxidant (entries 1 and 2), with very weak oxidant (entry 3), as well as with the dosage of external oxidant far less than the stoichiometric amount (entry 4).

When the reaction was conducted at room temperature under near-ultraviolet light irradiation from 365 nm Hg lamp (250 W), with 20 mol% commercial available anatase TiO_2_ NPs with a diameter of 60 nm as the photocatalyst, 6% yield was observed in the absence of external oxidant (entry 1). This reaction was accompanied by H_2_ gas formation, indicating that coupling of the photo-generated electrons with the protons released from decarboxylative C–H trifluoromethylation reaction occurred. The catalytic efficiency of the anatase TiO_2_ NPs could be enhanced by loading rhodium metal NPs of 0.1 wt % (relative to the amount of TiO_2_) onto their surface, which gave rise to 15% yield of trifluoromethylated benzene (entry 2). Interestingly, by employing 2 equivalences of Na_2_SO_4_ as additives, the reaction occurred smoothly to give the desired product in 51% yield (entry 3). Introduction of substoichiometric external oxidants, such as 0.2 equivalents of Na_2_S_2_O_8_ could increase the reaction yield to 70% (entry 4). The attempt to use visible light in place of ultraviolet light failed to give the satisfied result because of the wide band gap of TiO_2_ (entry 5). We also found that introducing one equivalence of CF_3_COONa into the reaction system almost shut down this reaction (entry 6), which presumably arose from the difference in substrate-TiO_2_ surface interaction between trifluoroacetic acid and CF_3_COONa. Moreover, control experiments revealed that both anatase TiO_2_ photocatalyst and ultraviolet light are necessary for the trifluoromethylation reaction to occur (entries 7 and 8).

### Substrate scope and gram-scale reations

**Figure 2:**
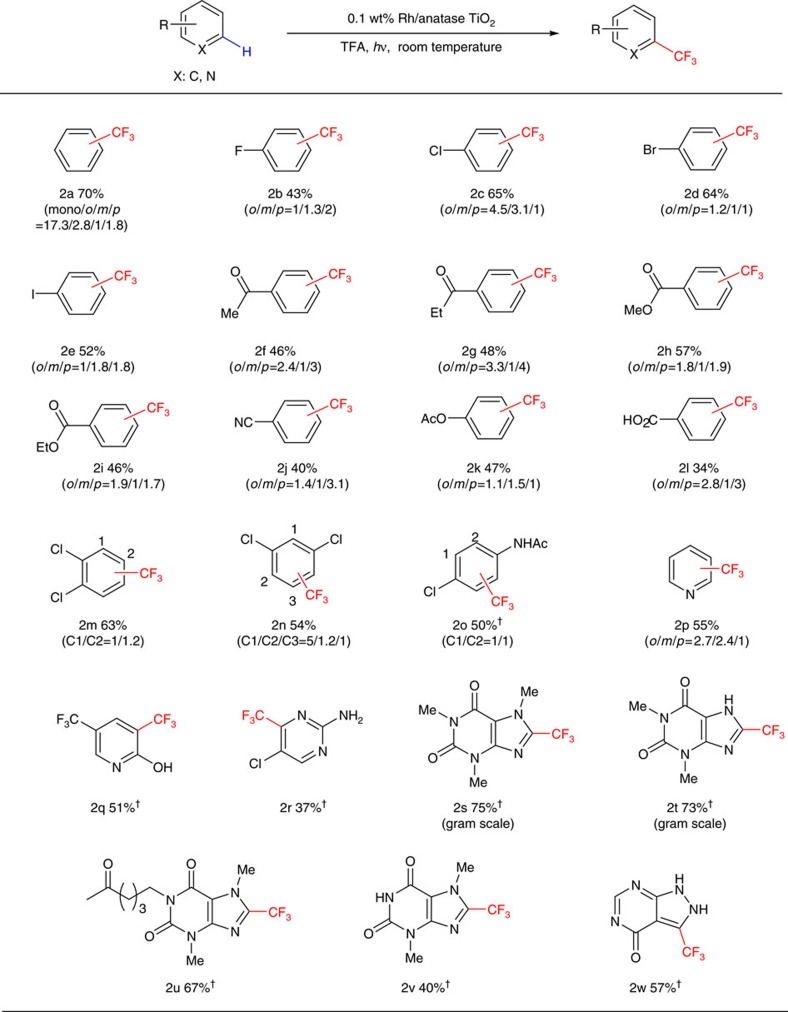
Photo-driven C–H trifluoromethylation of substituted benzenes and nitrogen-containing heteroarenes. Standard reaction conditions: substrates (0.5 mmol), 0.1 wt% Rh/anatase TiO_2_ NPs (20 mol%), Na_2_S_2_O_8_ (10-40 mol%), TFA (10–15 ml), 365 nm ultraviolet, room temperature, 24 h. Yields were determined by ^19^F NMR spectrum. ^†^Isolated yield.

With the established reaction conditions for the photo-driven decarboxylative C–H trifluoromethylation, we evaluated substrate scope of substituted benzenes in the presence of substoichiometric amount of oxidant (0.1–0.4 equivalences of Na_2_S_2_O_8_), using trifluoroacetic acid as a trifluoromethylating reagent (for more information, see Supplementary Methods and Supplementary Fig. 53). As shown in Fig. 2, this developed C–H trifluoromethylation reaction was compatible with a broad range of functional groups, such as halogen (**2b-2e**), keto (**2f**, **2g**), ester (**2h**, **2i**) and nitrile (**2j**) groups. Interestingly, ultraviolet light-sensitive C-I bond remained intact during the course of C–H trifluoromethylation (**2e**). And benzoic acid, which is prone to oxidative decarboxylation under oxidative conditions, could undergo trifluoromethylation reaction, albeit in a diminished yield (**2l**). Disubstituted benzenes (**2m**, **2n** and **2o**) could also be trifluoromethylated in synthetically useful yields. Moreover, a variety of nitrogen-containing heteroarenes, such as pyridines (**2p**, **2q**), pyrimidine (**2r**), pyrazolinopyrimidine (**2w**) and biologically active xanthines (**2s**-**2v**) were amenable to this trifluoromethylation reaction, affording a satisfying outcome. Importantly, the reactions of these nitrogen-containing heteroarenes exhibited a high level of position selectivity, and could be scaled up for the gram-scale synthesis as exemplified by the reactions of caffeine and theophylline (**2s**, **2t**), highlighting the practicality of this approach. In the established reaction, the use of 15 ml trifluoroacetic acid as a solvent may result from the need for sufficient light-absorbing surface to obtain satisfying yields presumably due to relative low concentration of catalytic active centres of heterogeneous TiO_2_-based nanocatalyst. Despite this, excess TFA in this reaction is reusable by means of easy distillation for its purification and collection.

**Figure 3:**
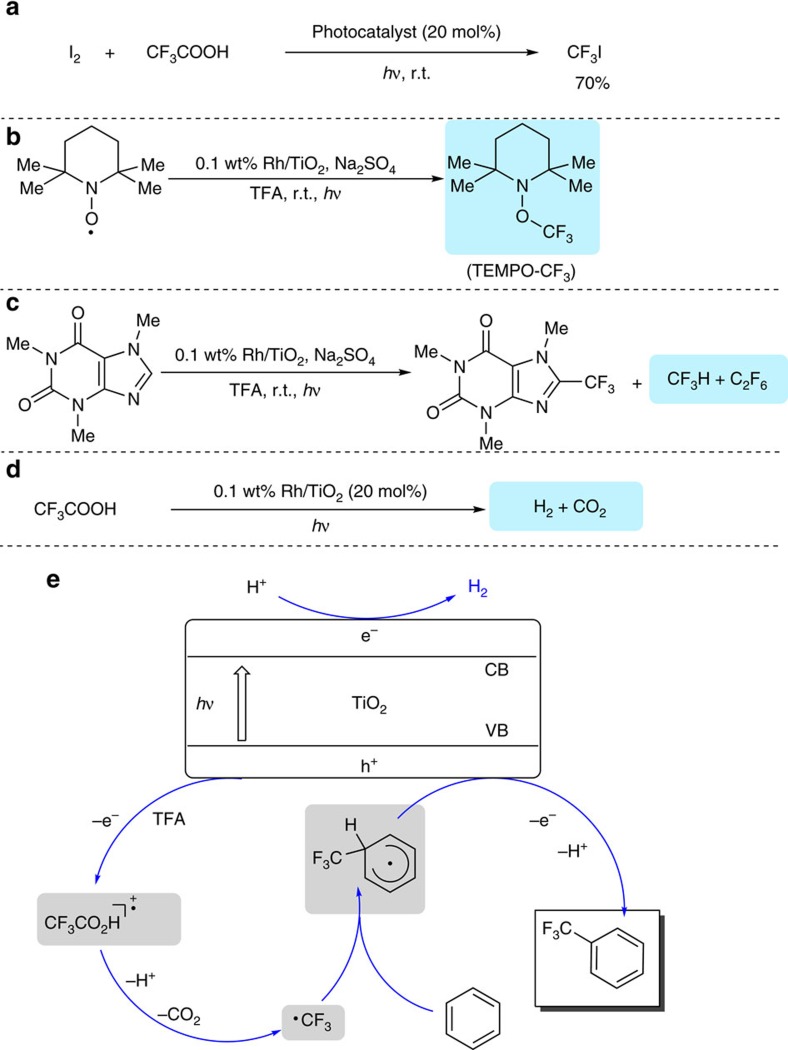
Mechanism studies. (**a**) Synthesis of CF_3_I via decarboxylative coupling of I_2_ with TFA. (**b**) Capture of CF_3_ radical by TEMPO. (**c**) Determination of CF_3_H and C_2_F_6_ side products in trifluoromethylation of caffeine. (**d**) Determination of H_2_ and CO_2_ from the photo-driven decarboxylation of TFA. (**e**) The proposed reaction mechanism.

The established reaction conditions for arene trifluoromethylation were applicable to the reaction of iodine with trifluoroacetic acid to afford trifluoromethyl iodide (CF_3_I), a commonly used trifluoromethylating reagent^13^, in a 70% yield (Fig. 3a and Supplementary Fig. 1), which provides an operationally simple approach to the efficient synthesis of CF_3_I.

### Mechanistic insights and nanocatalyst characterization

Although the position selectivity of this decarboxylative trifluoromethylation reaction suggested that the reaction would proceed via trifluoromethyl radical formation, further experiments were still required to gain insights into reaction mechanism. As shown in Fig. 3b, on adding 2,2,6,6-tetramethyl-1-piperidinyloxy (TEMPO), a well-known radical scavenger, to trifluoroacetic acid solution under standard photoredox conditions, the trifluoromethyl radicals generated from decarboxylation of trifluoroacetic acid was trapped with TEMPO to form TEMPO-CF_3_ adduct that was confirmed by ^19^F NMR spectroscopy (Supplementary Fig. 2).

In addition, both trifluoromethane (CF_3_H) and hexafluoroethane (C_2_F_6_) were also observed as side-products during the trifluoromethylation reaction of caffeine (Fig. 3c and Supplementary Figs 3 and 4), providing further evidence to support that trifluoromethyl radical is the intermediate of the decarboxylative trifluoromethylation reaction. Furthermore, formations of both H_2_ and CO_2_ were observed when trifluoroacetic acid was subjected to the standard reaction conditions in the absence of any arene reactant (Fig. 3d and Supplementary Figs 5 and 6), which is in accord with the aforementioned observation in the decarboxylative trifluoromethylation of benzene. The fact that the oxidative decarboxylation of trifluoroacetic acid is concurrent with H_2_ formation strongly suggested that the redox chemistry of the photo-driven water splitting to H_2_ and O_2_ operates in the decarboxylative C–H trifluoromethylation reaction with trifluoacetic acid as a trifluoromethyl source.

On the basis of mechanistic investigation, a possible reaction pathway was proposed for the TiO_2_-catalysed photo-driven decarboxylative trifluoromethylation with trifluoroacetic acid as trifluoromethyl source (Fig. 3e). Initially, the oxidation of trifluoroacetic acid by the photo-generated hole in the valence band of TiO_2_ leads to rapid decarboxylation to form trifluoromethyl radical with carbon dioxide and proton released. The resultant trifluoromethyl radical is then intercepted by benzene to produce trifluoromethyl benzene radial. Finally, this trifluoromethyl benzene radical undergoes oxidation through a single-electron transfer process and subsequent deprotonation to afford a trifluoromethylated benzene. Meanwhile, the released protons accept the photo-generated electron to release hydrogen gas, a process similar to the photo-driven water splitting to H_2_ evolution^30^. Alternatively, the external oxidant introduced into the reaction system can also accept the photo-generated electron to drive the reaction. Consequent, combination of contributions of proton and a substoichiometric amount of external oxidant provided good yields for the photo-driven decarboxylative trifluoromethylation reaction.

**Figure 4:**
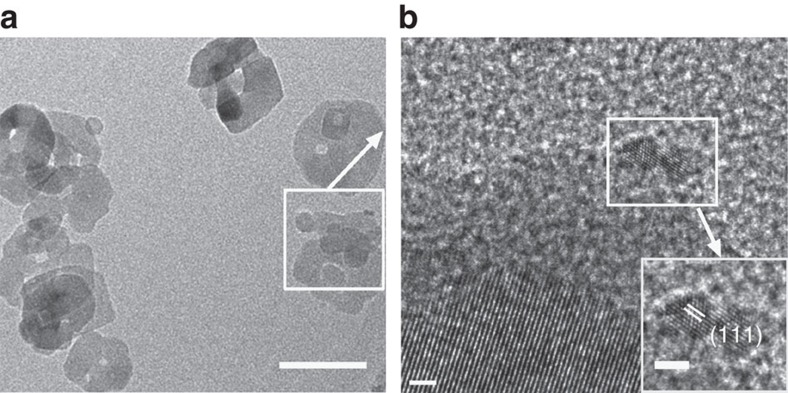
Characterization of the nanocatalyst. (**a**) TEM image of Rh/TiO_2_ (scale bar, 50 nm), (**b**) HRTEM image (scale bar, 5 nm) and lattice fringe image (inset, scale bar of 2 nm) of Rh(0) NP. HRTEM, high-resolution TEM.

The photocatalyst used in this reaction, that is, the Rh-modified TiO_2_ NPs, were structurally characterized. The inductively coupled plasma mass spectrometry revealed that this Rh/TiO_2_ photocatalyst contained Rh content of 0.1 wt%. The powder X-ray diffraction pattern of this nanocatalyst could be exclusively indexed to anatase-phase TiO_2_ (Supplementary Fig. 7). The transmission electron microscopy (TEM) image (Fig. 4a) and high-resolution TEM image (Fig. 4b) showed that the metallic Rh NPs with an average size of 2.04 nm (Supplementary Figs 8–10) was located on the surface of anatase NPs of about 60 nm. Besides, X-ray photoelectron spectroscopy indicated the signals of Rh 3d_5/2_ and 3d_3/2_ with binding energies of 306.42 and 311.27 eV, typical of Rh (0), further verifying that Rh existed in the form of metallic Rh (Supplementary Figs 11–15)^31^.

## Discussion

On the basis of the characterization of the Rh-modified anatase TiO_2_ NPs, we attempt to rationalize the photocatalysis efficiency enhancement associated with the attachment of metallic Rh NPs onto the surface of anatase TiO_2_ NPs. Presumably, in the Rh-modified anatase NPs, the difference in Fermi levels between metal Rh and semiconductor TiO_2_ may give rise to the migration of photo-generated electron from TiO_2_ with higher Fermi level (the work function of TiO_2_ is 4.8 eV) to Rh with lower Fermi level (the work function of Rh is 5.6 eV) until equilibrium alignment of two Femi levels^32^. As a result, the Schottky barrier is created at the interface between Rh and TiO_2_ NPs^33^. Such a barrier may act as an effective trap for electron capture, and prevent from recombination between photo-generated electron/hole pairs, which eventually leads to the improvement of catalytic efficiency (shown in Supplementary Fig. 16).

In this work, we have developed a photo-driven C–H trifluoromethylation reaction that enables the use of readily available and cheap trifluoroacetic acid as a trifluoromethyl source for high-valuable trifluoromethylation reactions. Importantly, such a photo-driven reaction could proceed with the dosage of external oxidant far less than the stoichiometric amount or even without any external oxidant, in which H_2_ release provided an essential driven force for reaction to occur. This reaction demonstrates that the principle underlying the photo-driven water splitting to H_2_ and O_2_ is applicable to C–H functionalization reaction, which will inspire the development of photocatalysts to improve efficiencies of both utility of light and coupling of the proton from C–H activation process with the photo-generated electron and ultimately achieve general approach to external oxidant-free C–H functionalization reactions^34^.

## Methods

### Preparation of 0.1 wt% Rh-modified anatase TiO_2_ nanopaticles

In a 30 ml quartz round bottom flask, a mixture of 60 nm anatase TiO_2_ (Aladdin reagent, 3.0 g) and RhCl_3_·xH_2_O (0.023 g, Rh content of 39%) was deoxygenated and filled with N_2_, followed by addition of deoxygenated methanol (30 ml). The suspension was stirred with an irradiation of 250 W high-pressure Hg lamp (365 nm ultraviolet) for 48 h. After illumination, nanocatalysts were separated from the solution by centrifugation (8,000 r.p.m., 2 min, 298 K). The separated nanocatalysts were washed for three times by ethanol and three times by water, dried in vacuum at 298 K for 8 h. The Rh content of as-prepared nanocatalyst was 0.1 wt% by inductively coupled plasma analysis.

### General procedure for photo-driven C–H trifluoromethylation

The photo-driven C–H trifluoromethylation reaction of caffeine is used as an example. Photocatalyst (0.016 g, 0.2 mmol), caffeine (0.0971, g, 0.5 mmol) and Na_2_S_2_O_8_ (0.0238, g, 0.1 mmol) were introduced into a Schlenk tube. Then, the tube was fitted with a rubber septum. After evacuation and N_2_ backfill for three times, distilled trifluoroacetic acid (15 ml) was added to the Schlenk tube through the rubber septum using syringes, and the rubber septum was replaced by a Teflon cap under N_2_ flow. The reaction was performed under illumination of 250 W high-pressure Hg lamp (365 nm ultraviolet) at room temperature for 48 h. After reaction, the trifluoroacetic acid was removed under reduced pressure and the residue was purified by flash chromatography on silica gel (eluent: EtOAc/hexanes) to provide the corresponding product. The yield were also determined by ^19^F NMR spectrum using 1-methoxy-4-(trifluoromethoxy)benzene (76 μl, 0.5 mmol, δ–58.4 p.p.m.) as an internal reference. For NMR spectra of the compounds in this article, see Supplementary Figs 17–52.

### Data availability

The data supporting this study are available from the corresponding author upon reasonable request.

## Additional information

**How to cite this article:** Lin, J. *et al*. Photo-driven redox-neutral decarboxylative carbon-hydrogen trifluoromethylation of (hetero)arenes with trifluoroacetic acid. *Nat. Commun.* **8,** 14353 doi: 10.1038/ncomms14353 (2017).

**Publisher's note:** Springer Nature remains neutral with regard to jurisdictional claims in published maps and institutional affiliations.

## Supplementary Material

Supplementary Information Supplementary Figures, Supplementary Table, Supplementary Methods and Supplementary References

Peer Review File

## Figures and Tables

**Table 1:**
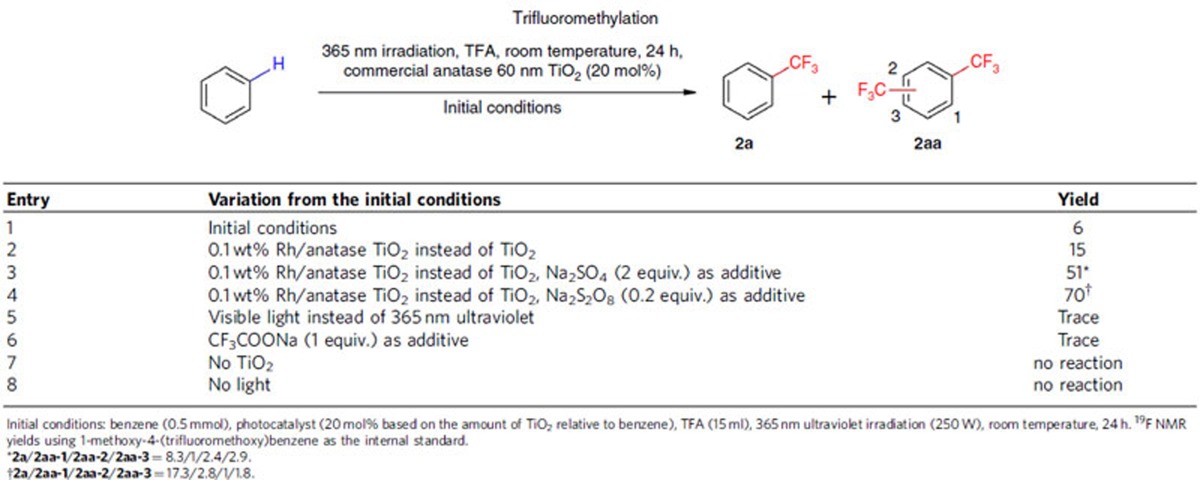
Effects of reaction parameters on photo-driven decarboxylative C–H trifluoromethylation.
