# Supplementary material for: Photo-driven redox-neutral decarboxylative carbon-hydrogen trifluoromethylation of (hetero)arenes with trifluoroacetic acid
Source: Nat Commun. 2017 Feb 6;8:14353. doi: 10.1038/ncomms14353 (PMC5303830; doi:10.1038/ncomms14353)
Supplement: Supplementary Information — Supplementary Figures, Supplementary Table, Supplementary Methods and Supplementary References [file ncomms14353-s1.pdf]

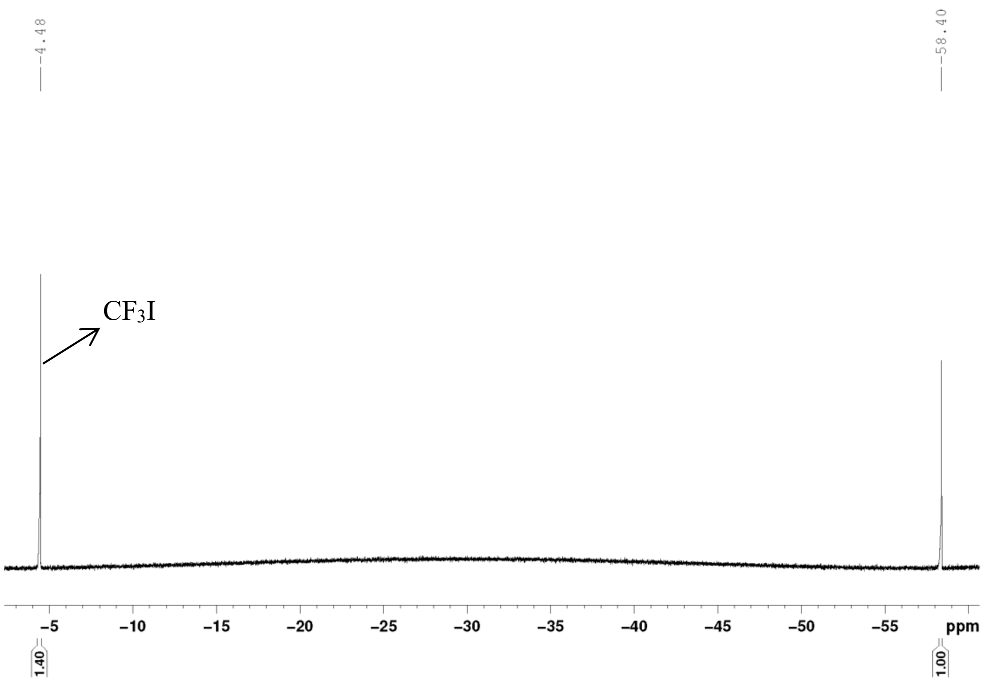

Supplementary Figure 1. The production of CF_3I from the reaction of trifluoroacetic acid with I_2

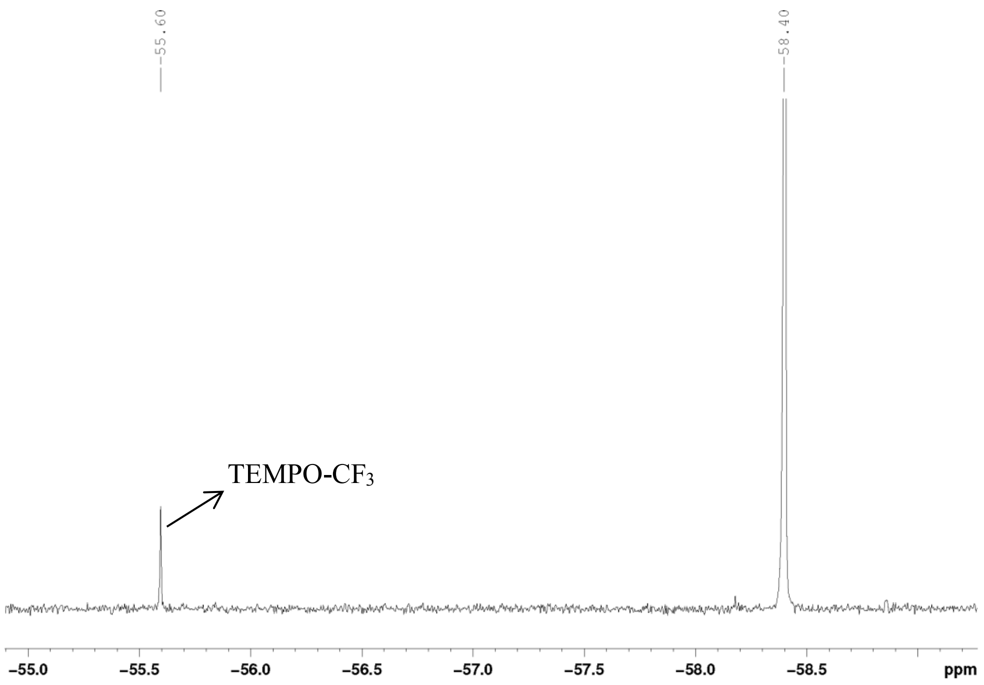

Supplementary Figure 2. The detection of TEMPO-CF_3 by ^{19}F NMR

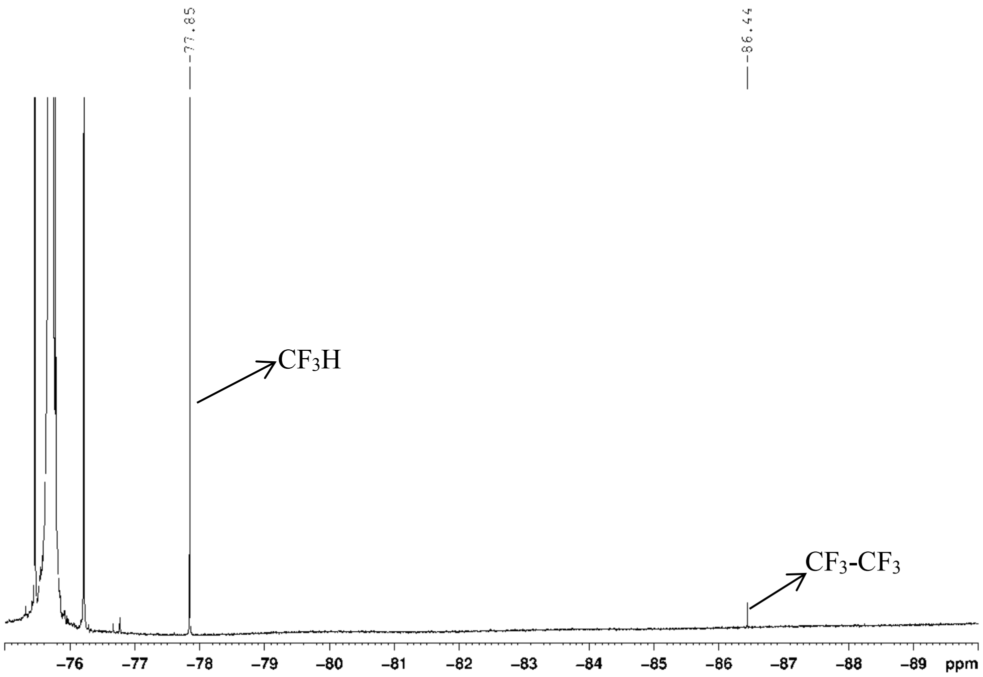

Supplementary Figure 3. ^{19}F-decoupled NMR spectrum for observation of trifluoromethane (CF_3H) and hexafluoroethane (CF_3-CF_3) by-products

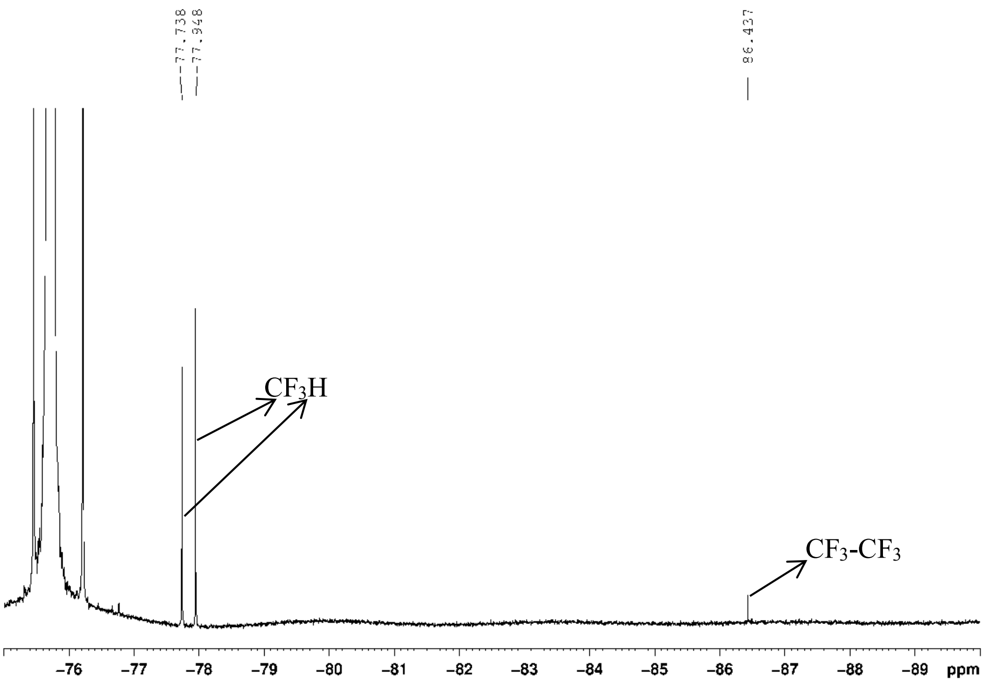

Supplementary Figure 4. ^{19}F-coupled NMR spectrum for observation of trifluoromethane (CF_3H) and hexafluoroethane (CF_3-CF_3)

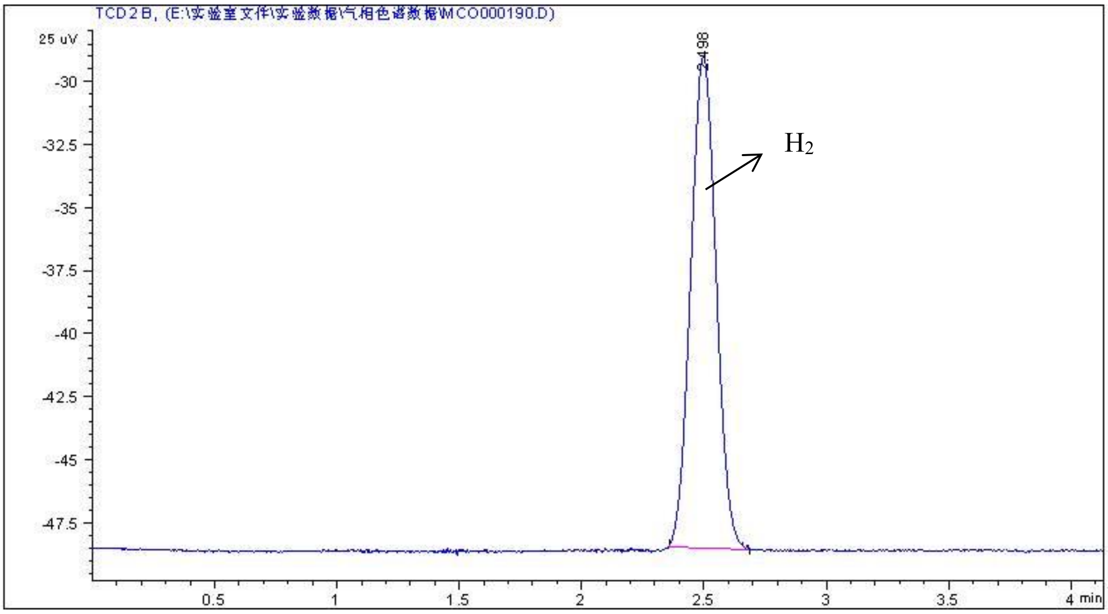

Supplementary Figure 5. The determination of H_2 by GC

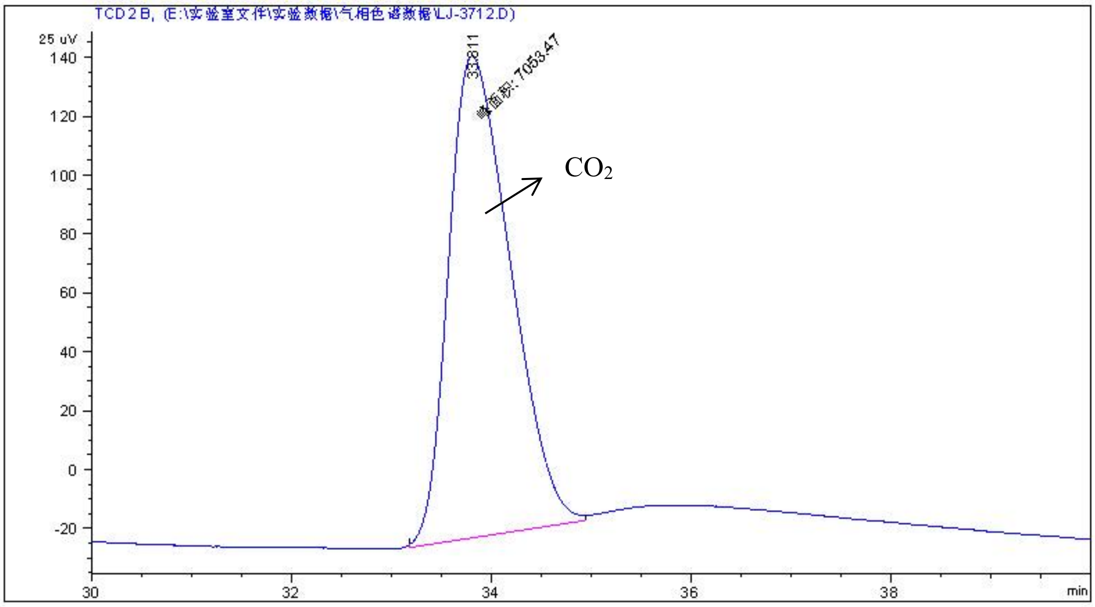

Supplementary Figure 6. The determination of CO_2 by GC

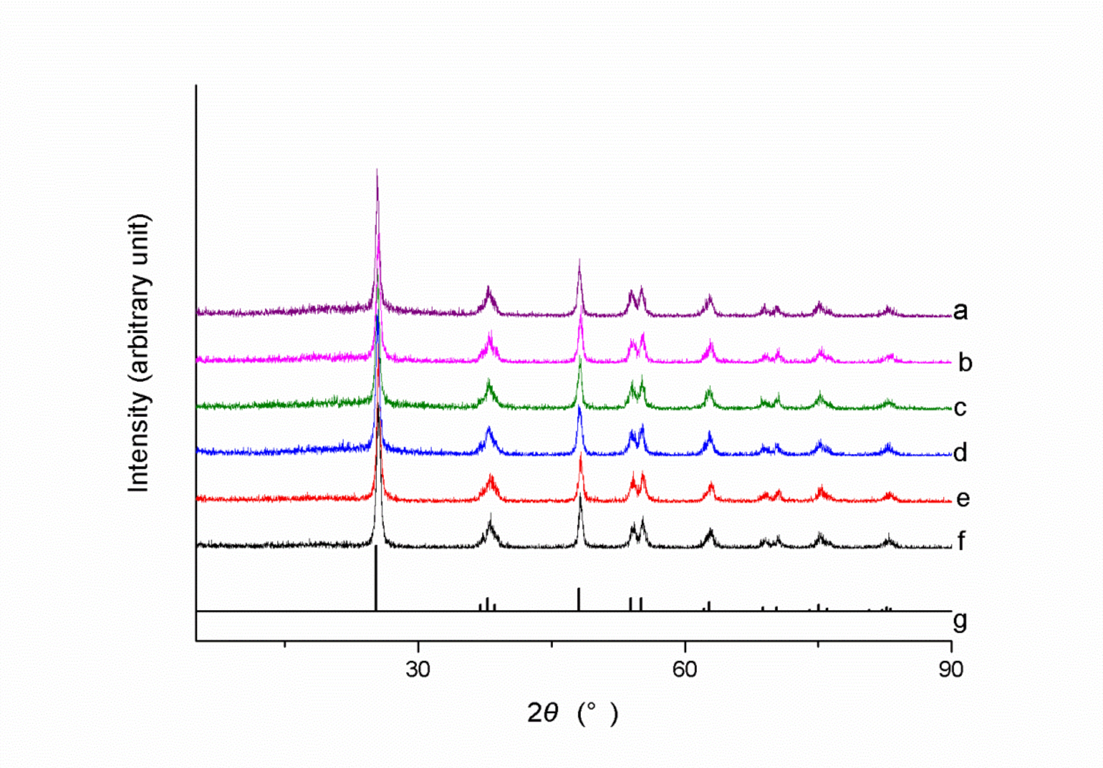

Supplementary Figure 7. XRD patterns of anatase TiO_2 nanocatalyst supported with different metals. (a) Rh/anatase TiO_2, (b) Ag/anatase TiO_2, (c) Pt/anatase TiO_2, (d) Cu/anatase TiO_2, (e) Au/anatase TiO_2, (f) Pd/anatase TiO_2, (g) standard profile of anatase TiO_2 PDF No. 21-1272.

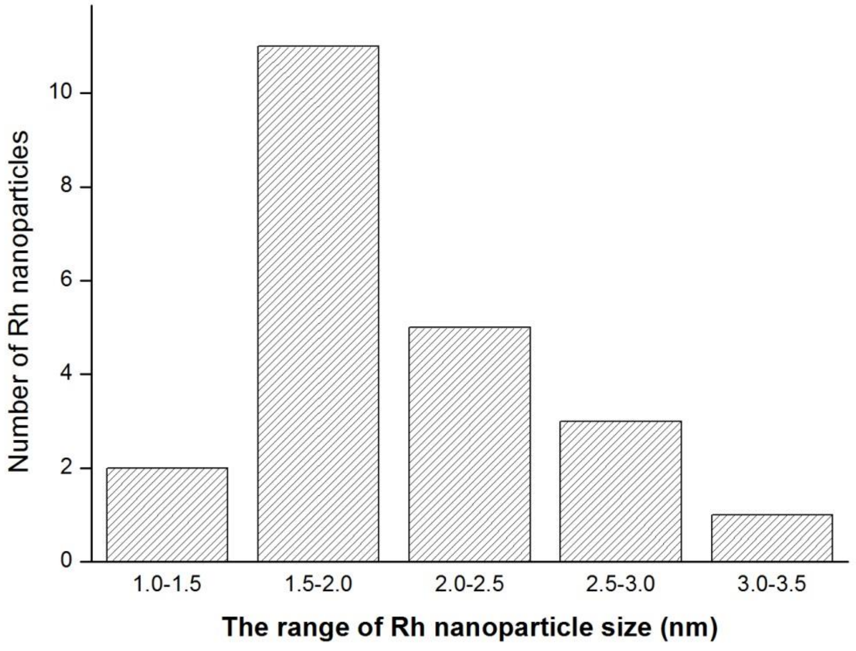

Supplementary Figure 8. the particle size distribution of Rh(0) nanoparticles on anatase TiO_2 nanocatalyst

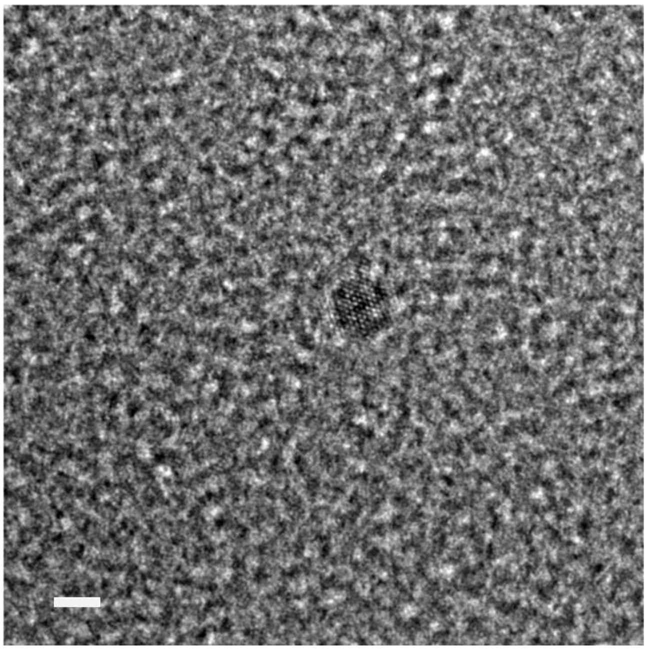

Supplementary Figure 9. HRTEM image of Rh/anatase TiO_2 nanocatalyst after reaction. (The scale bar, 2 nm)

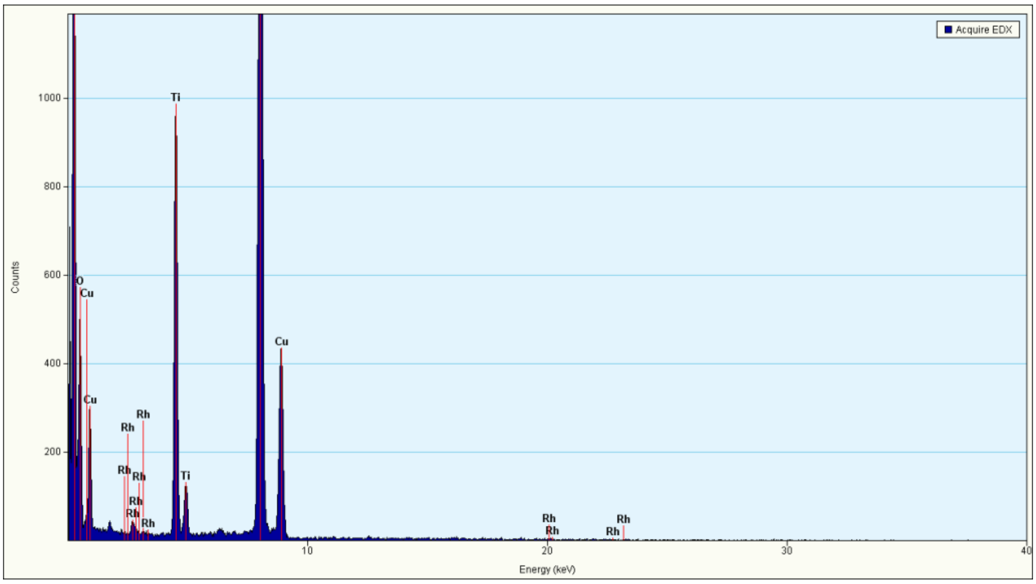

Supplementary Figure 10. EDS diagram of as prepared Rh/anatase TiO_2 nanocatalyst

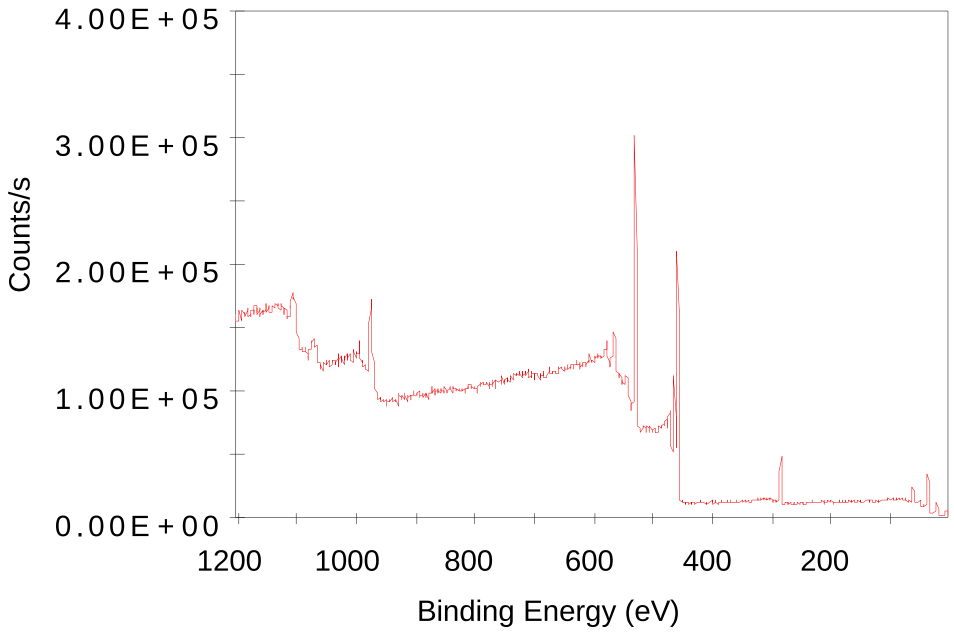

Supplementary Figure 11. XPS full spectrum of Rh supported anatase TiO_2

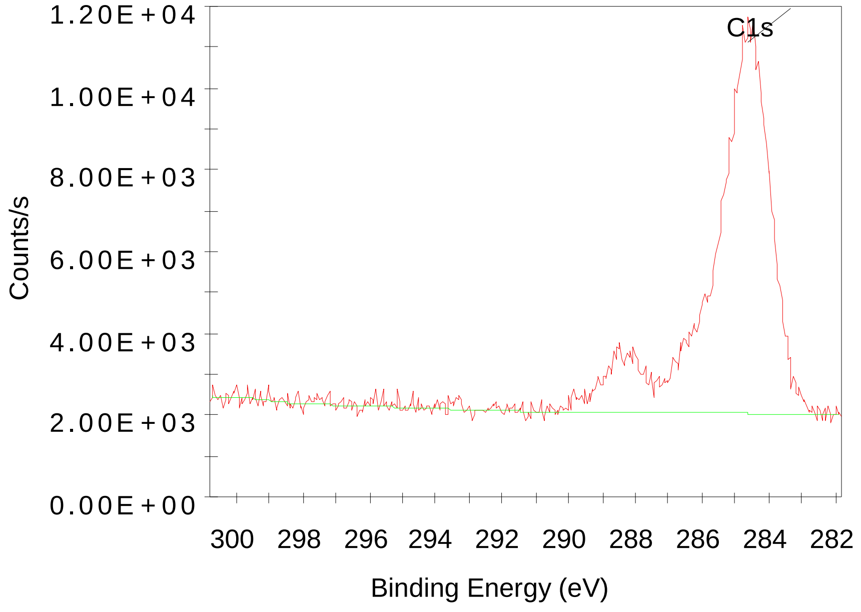

Supplementary Figure 12. XPS C_{1s} scanning spectrum of Rh supported anatase TiO_2

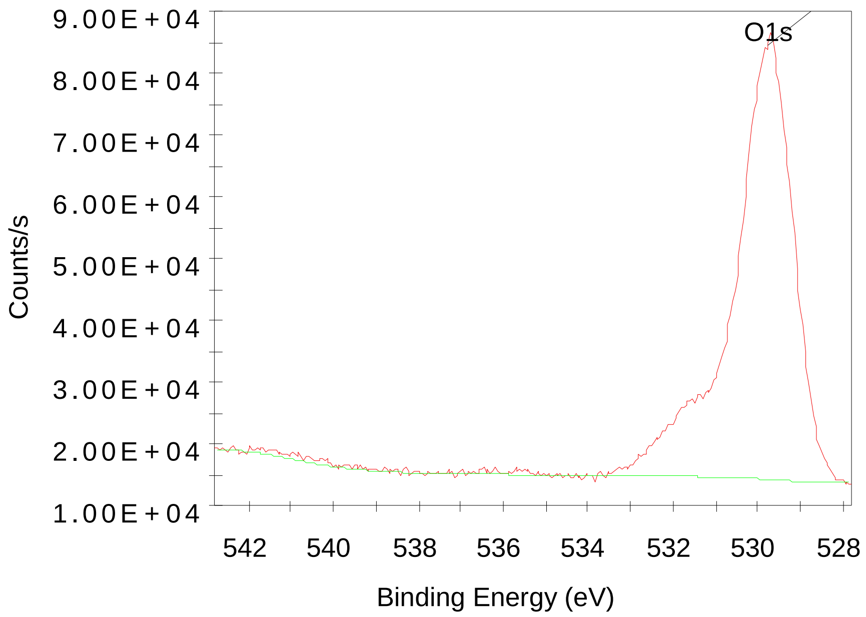

Supplementary Figure 13. XPS O_{1s} scanning spectrum of Rh supported anatase TiO_2

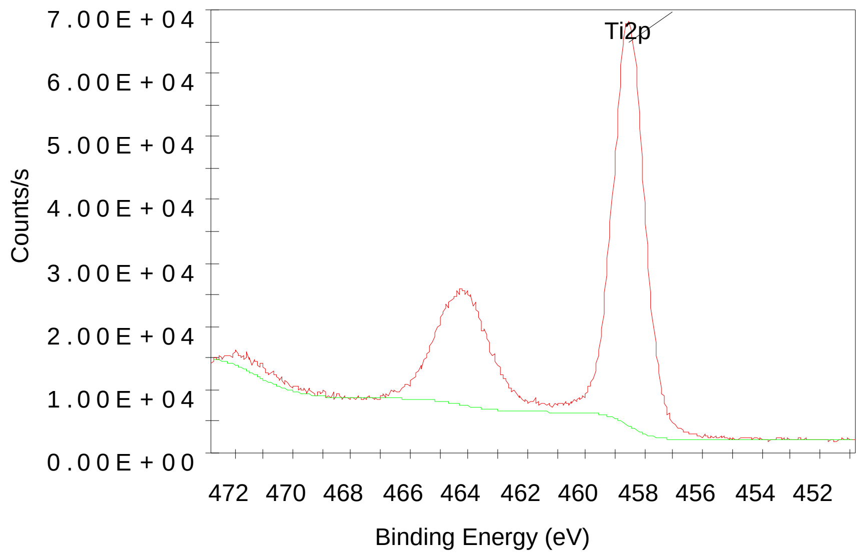

Supplementary Figure 14. XPS Ti_{2p} scanning spectrum of Rh supported anatase TiO_2

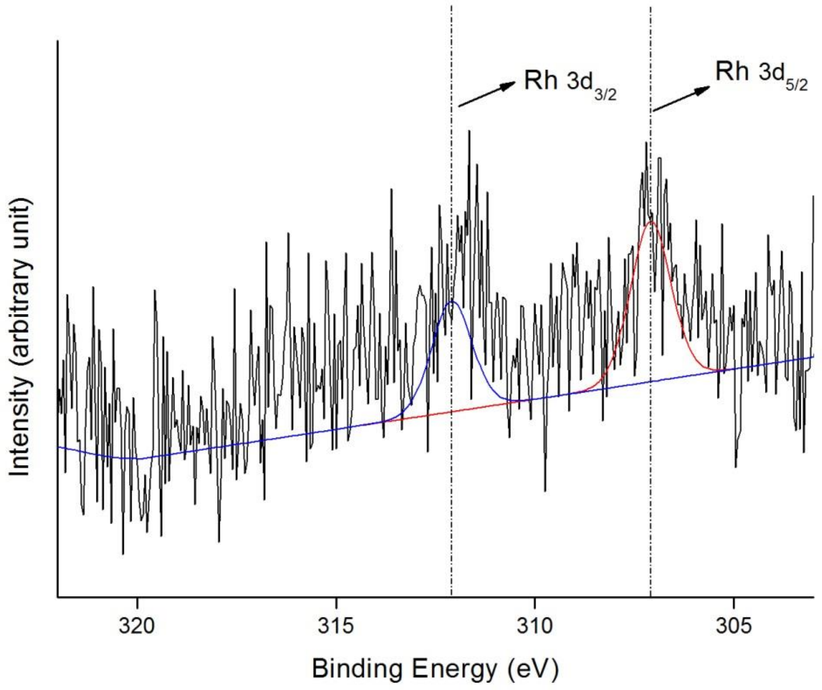

Supplementary Figure 15. XPS Rh_{3d} scanning spectrum of Rh supported anatase TiO_2

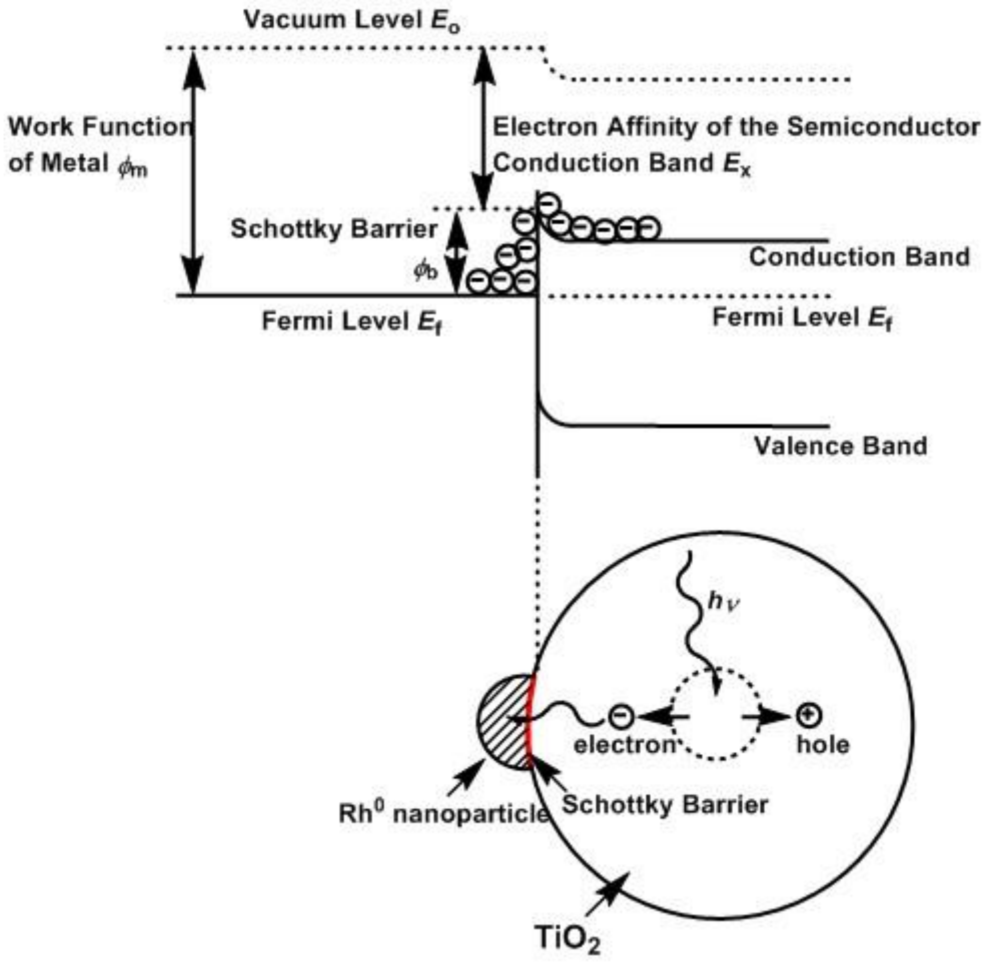

Supplementary Figure 16. Schottky barrier at the interface between Rh and TiO_2

N-(4-chloro-3-(trifluoromethyl)phenyl)acetamide (**2o**-C1)

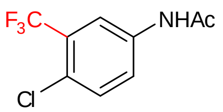

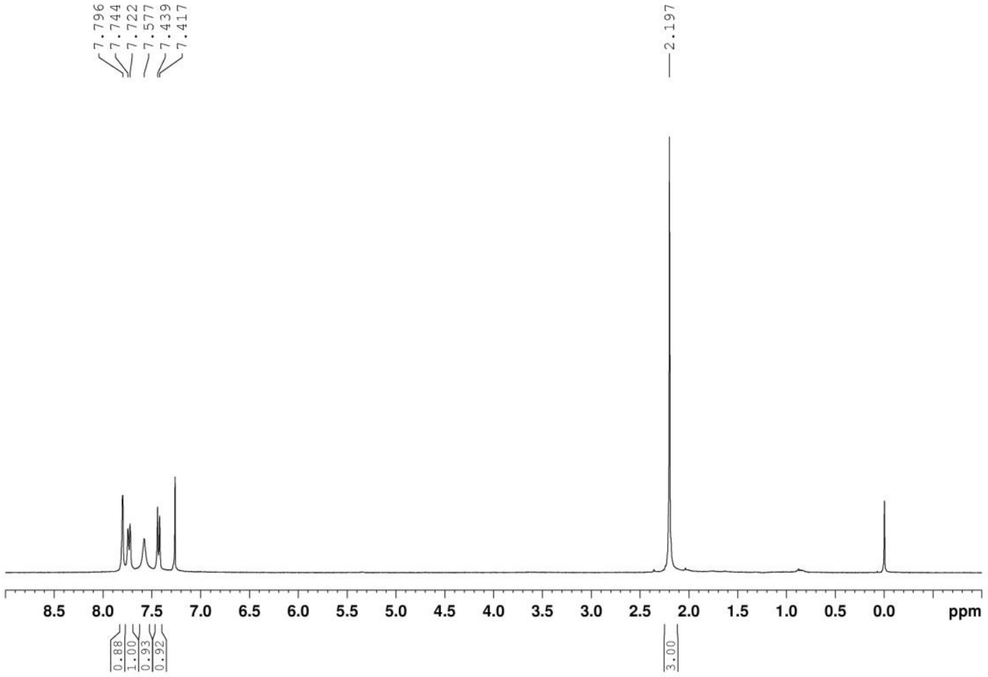

Supplementary Figure 17. ^{1}H NMR spectrum of N-(4-chloro-3-(trifluoromethyl)phenyl)acetamide (**2o**-C1)

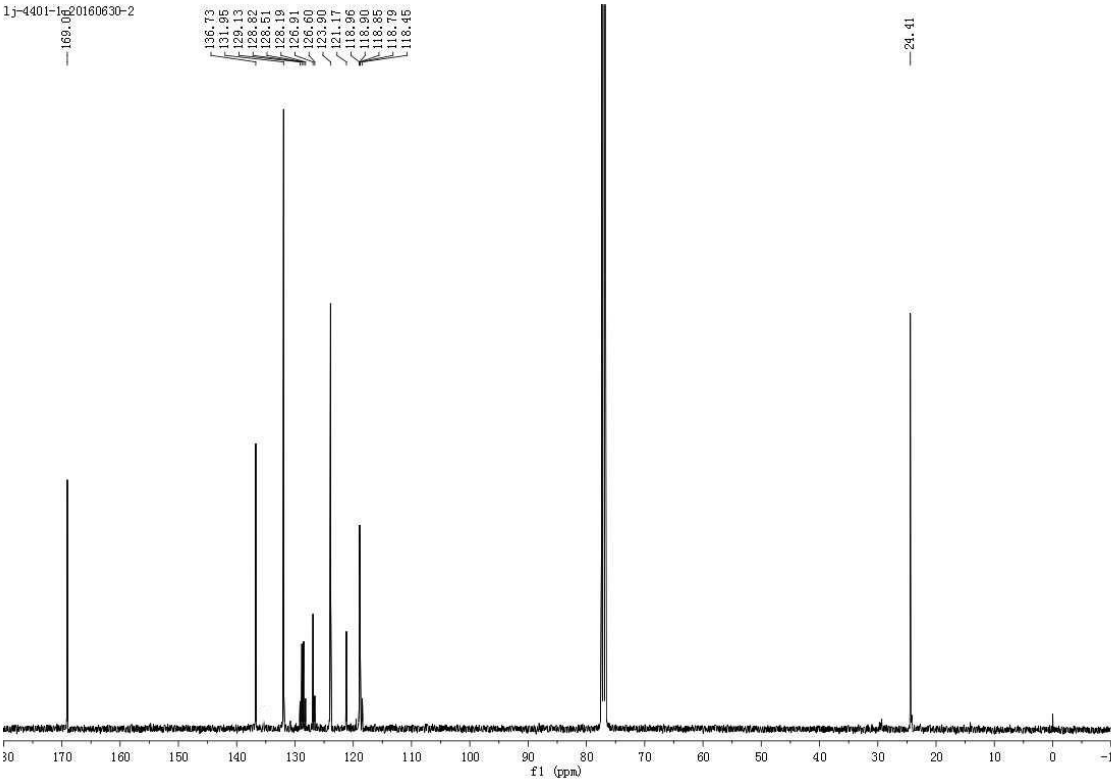

Supplementary Figure 18. ^{13}C NMR spectrum of N-(4-chloro-3-(trifluoromethyl)phenyl)acetamide (**2o**-C1)

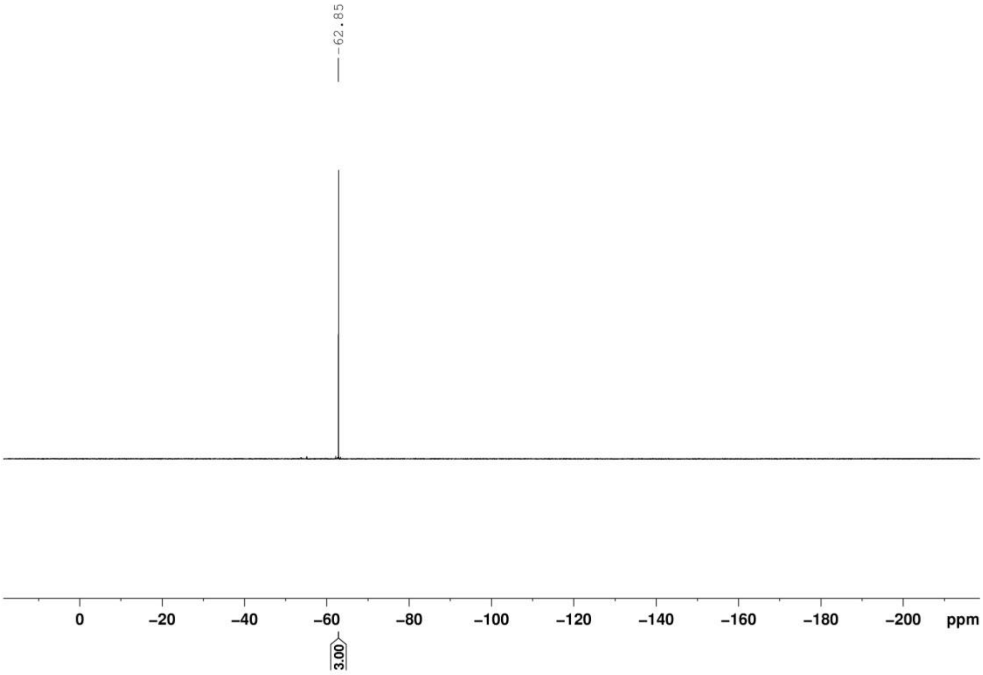

Supplementary Figure 19. ^{19}F NMR spectrum of N-(4-chloro-3-(trifluoromethyl)phenyl)acetamide (**2o**-C1)

N-(4-chloro-2-(trifluoromethyl)phenyl)acetamide (**2o**-C2)

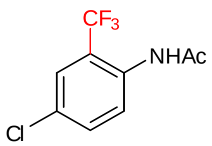

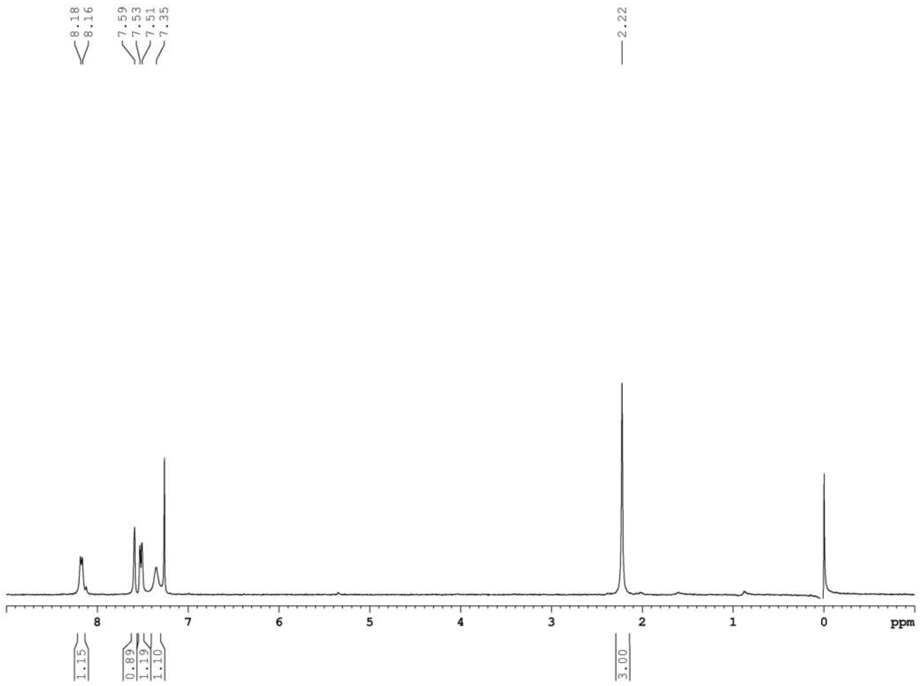

Supplementary Figure 20. ^{1}H NMR spectrum of N-(4-chloro-2-(trifluoromethyl)phenyl)acetamide (**2o**-C2)

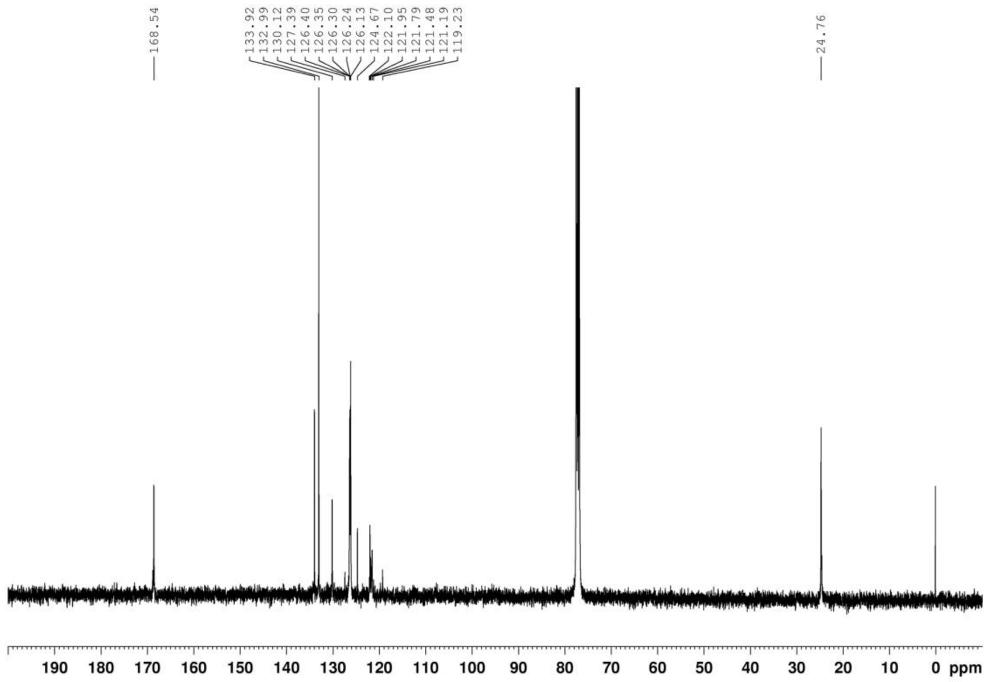

Supplementary Figure 21. ^{13}C NMR spectrum of N-(4-chloro-2-(trifluoromethyl)phenyl)acetamide (**2o**-C2)

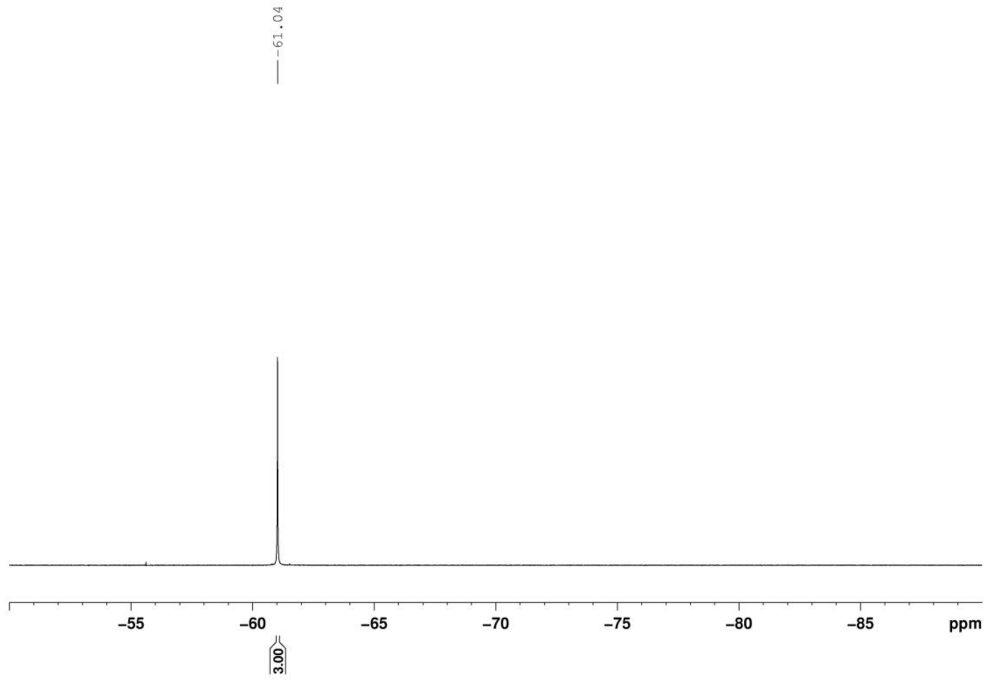

Supplementary Figure 22. ^{19}F NMR spectrum of N-(4-chloro-2-(trifluoromethyl)phenyl)acetamide (**2o**-C2)

3,5-bis(trifluoromethyl)pyridin-2-ol **(2q)**

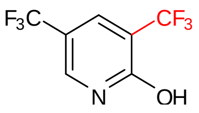

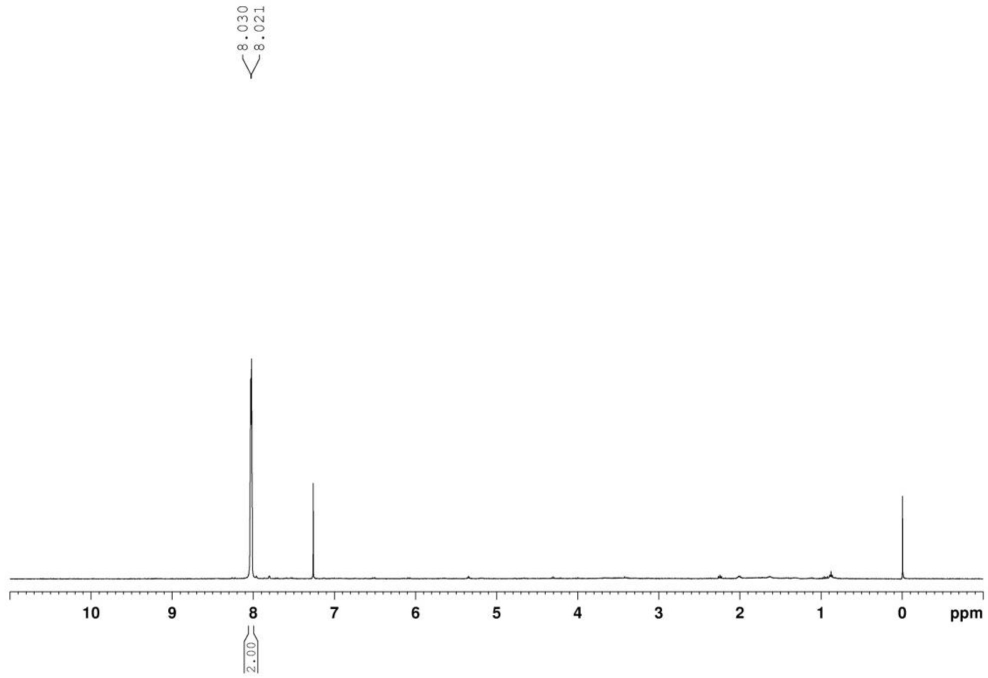

Supplementary Figure 23. ^{1}H NMR spectrum of 3,5-bis(trifluoromethyl)pyridin-2-ol (**2q**)

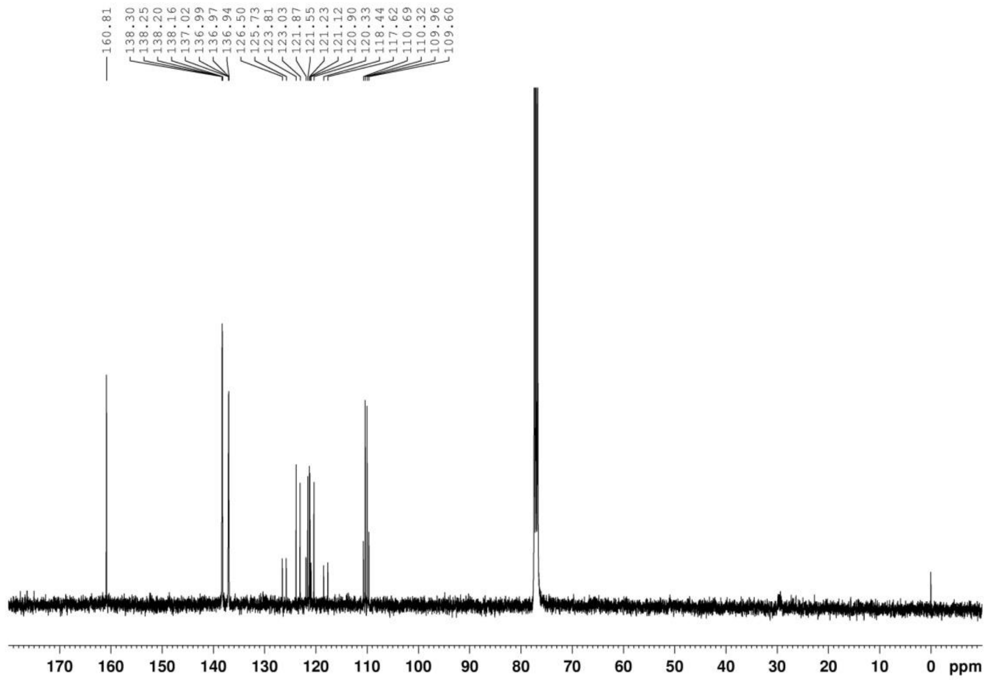

Supplementary Figure 24. ^{13}C NMR spectrum of 3,5-bis(trifluoromethyl)pyridin-2-ol (**2q**)

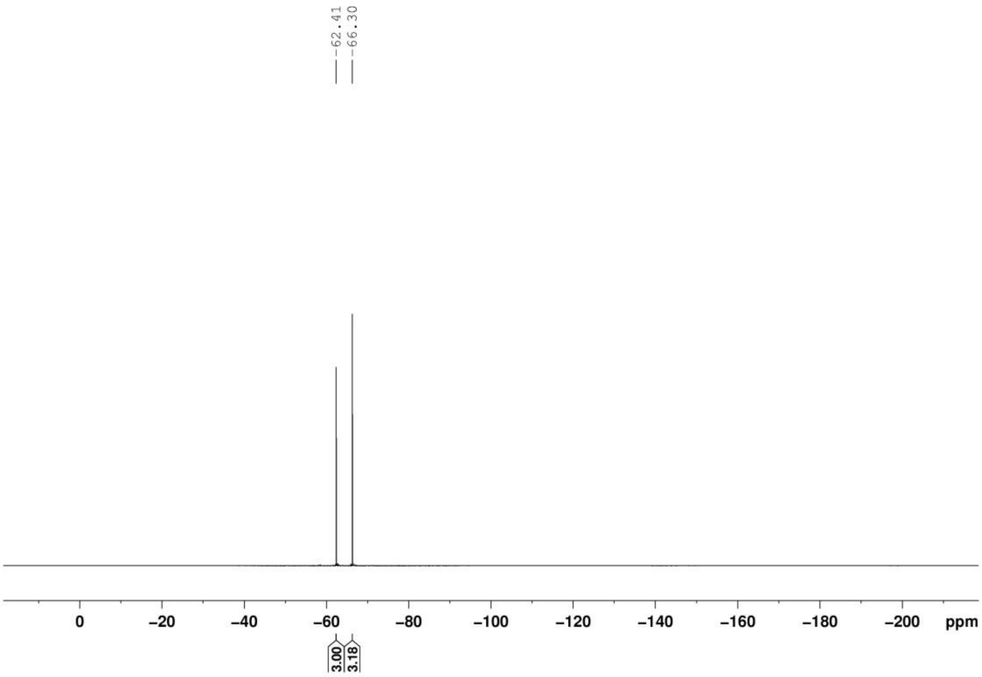

Supplementary Figure 25. ^{19}F NMR spectrum of 3,5-bis(trifluoromethyl)pyridin-2-ol (**2q**)

5-Chloro-4-(trifluoromethyl)-2-pyrimidinamine (**2r**)

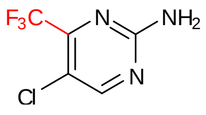

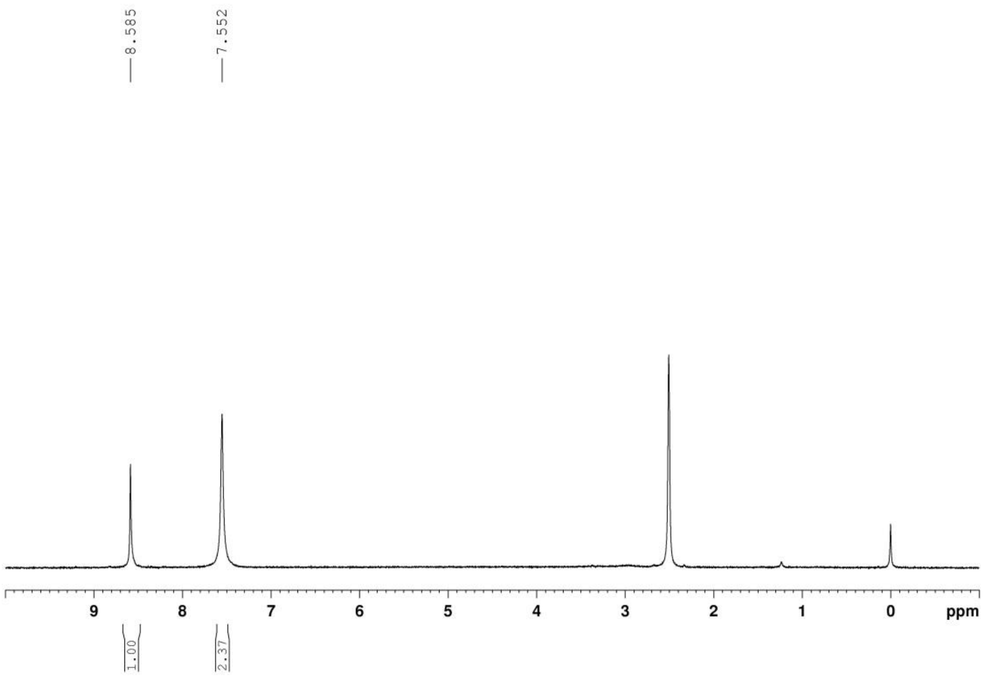

Supplementary Figure 26. ^{1}H NMR spectrum of 5-Chloro-4-(trifluoromethyl)-2-pyrimidinamine (**2r**)

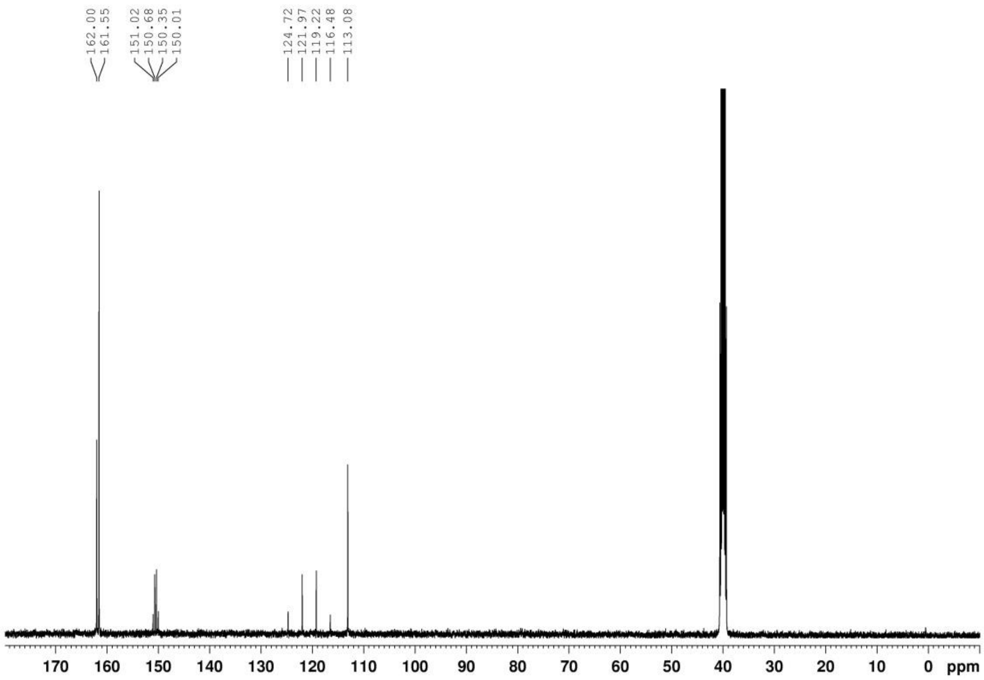

Supplementary Figure 27. ^{13}C NMR spectrum of 5-Chloro-4-(trifluoromethyl)-2-pyrimidinamine (**2r**)

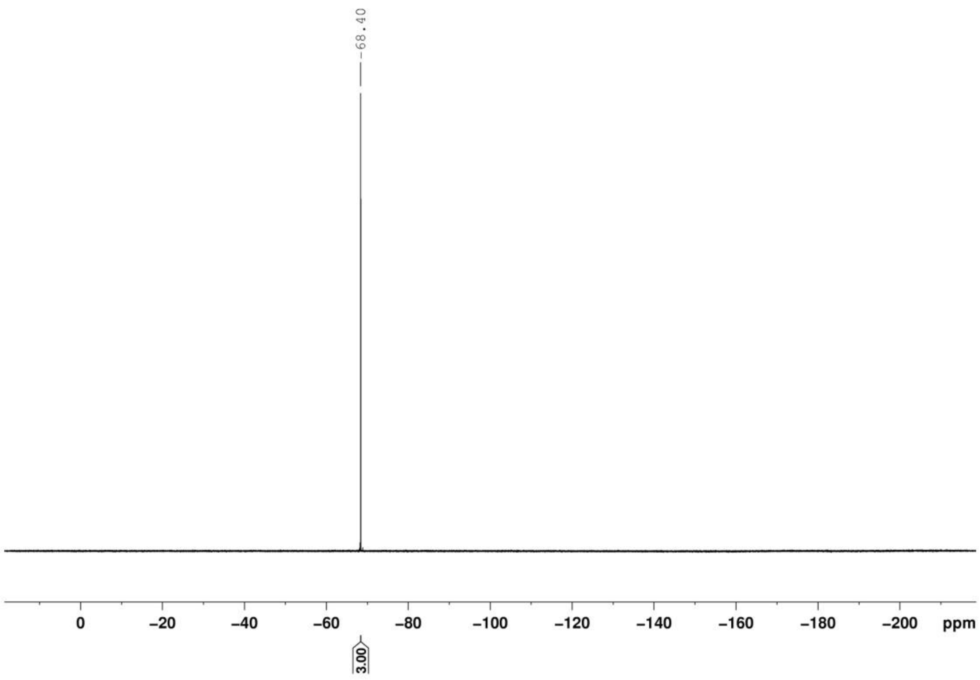

Supplementary Figure 28. ^{19}F NMR spectrum of 5-Chloro-4-(trifluoromethyl)-2-pyrimidinamine (**2r**)

1,3,7-trimethyl-8-(trifluoromethyl)-1*H*-purine-2,6(3*H*,7*H*)-dione (**2s**)

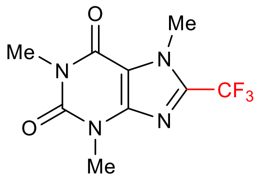

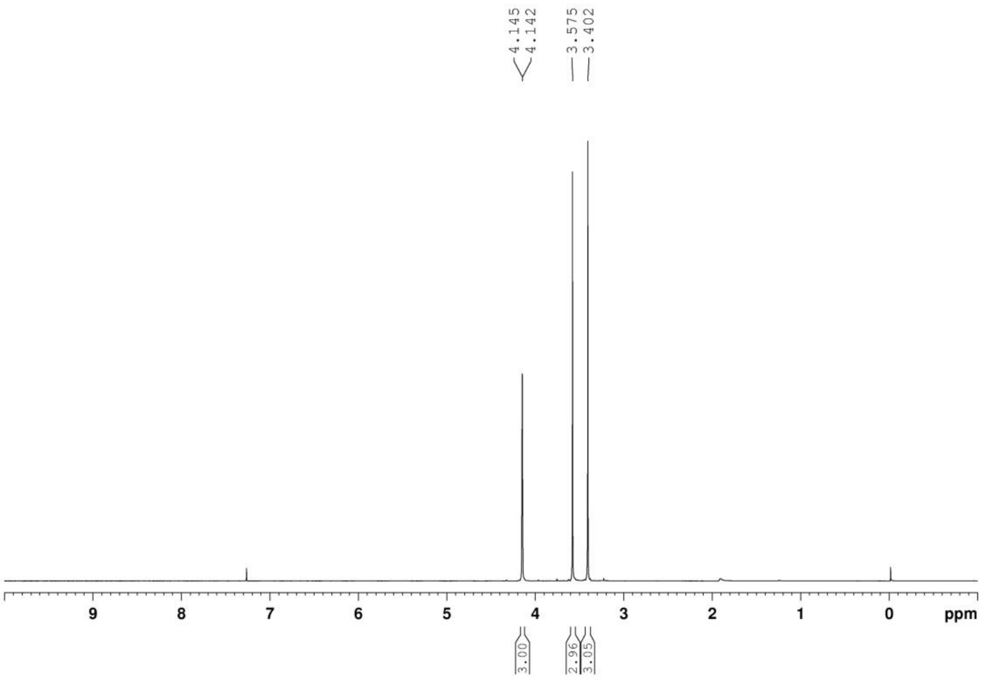

Supplementary Figure 29. ^{1}H NMR spectrum of 1,3,7-trimethyl-8-(trifluoromethyl)-1*H*-purine-2,6(3*H*,7*H*)-dione (**2s**)

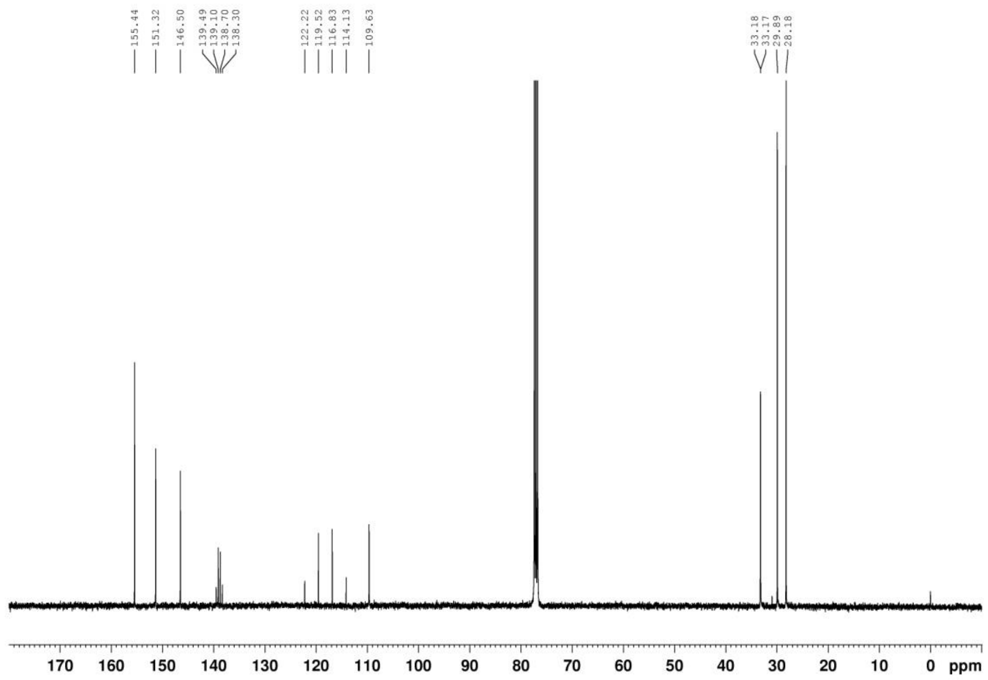

Supplementary Figure 30. ^{13}C NMR spectrum of 1,3,7-trimethyl-8-(trifluoromethyl)-1*H*-purine-2,6(3*H*,7*H*)-dione (**2s**)

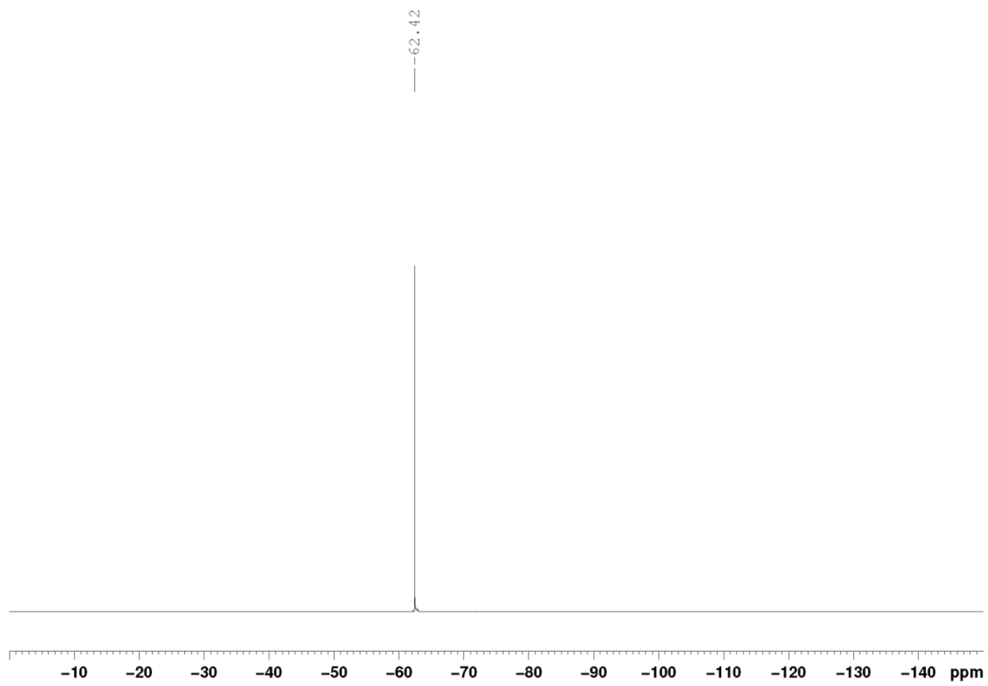

Supplementary Figure 31. ^{19}F NMR spectrum of 1,3,7-trimethyl-8-(trifluoromethyl)-1*H*-purine-2,6(3*H*,7*H*)-dione (**2s**)

1,3-dimethyl-8-(trifluoromethyl)-1*H*-purine-2,6(3*H*,7*H*)-dione (**2t**)

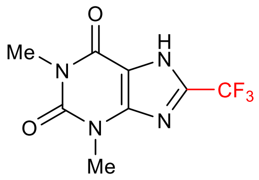

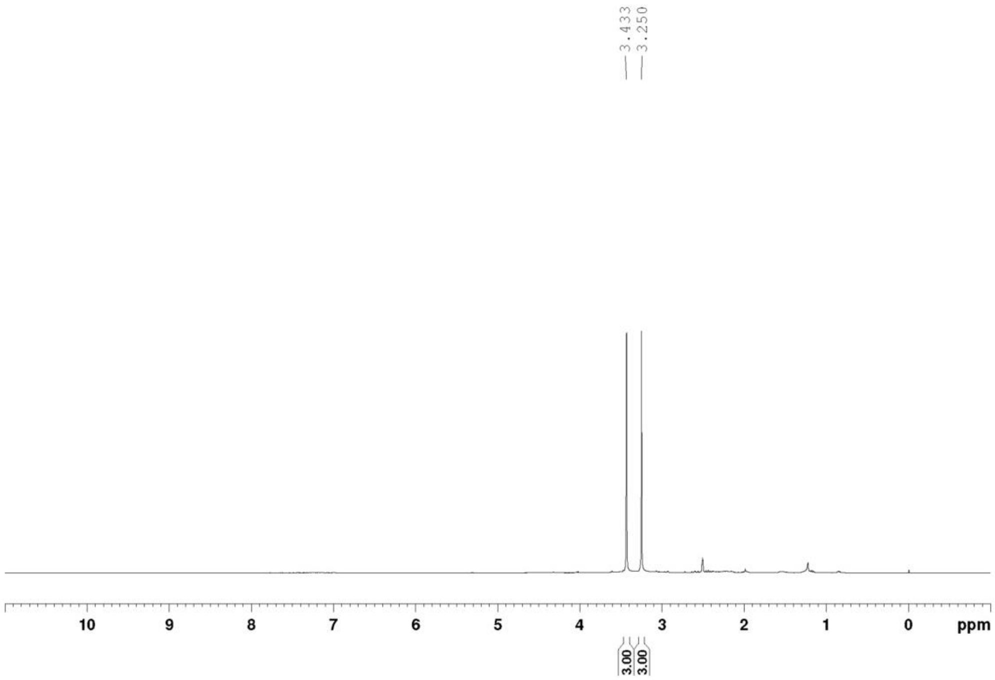

Supplementary Figure 32. ^{1}H NMR spectrum of 1,3-dimethyl-8-(trifluoromethyl)-1*H*-purine-2,6(3*H*,7*H*)-dione (**2t**)

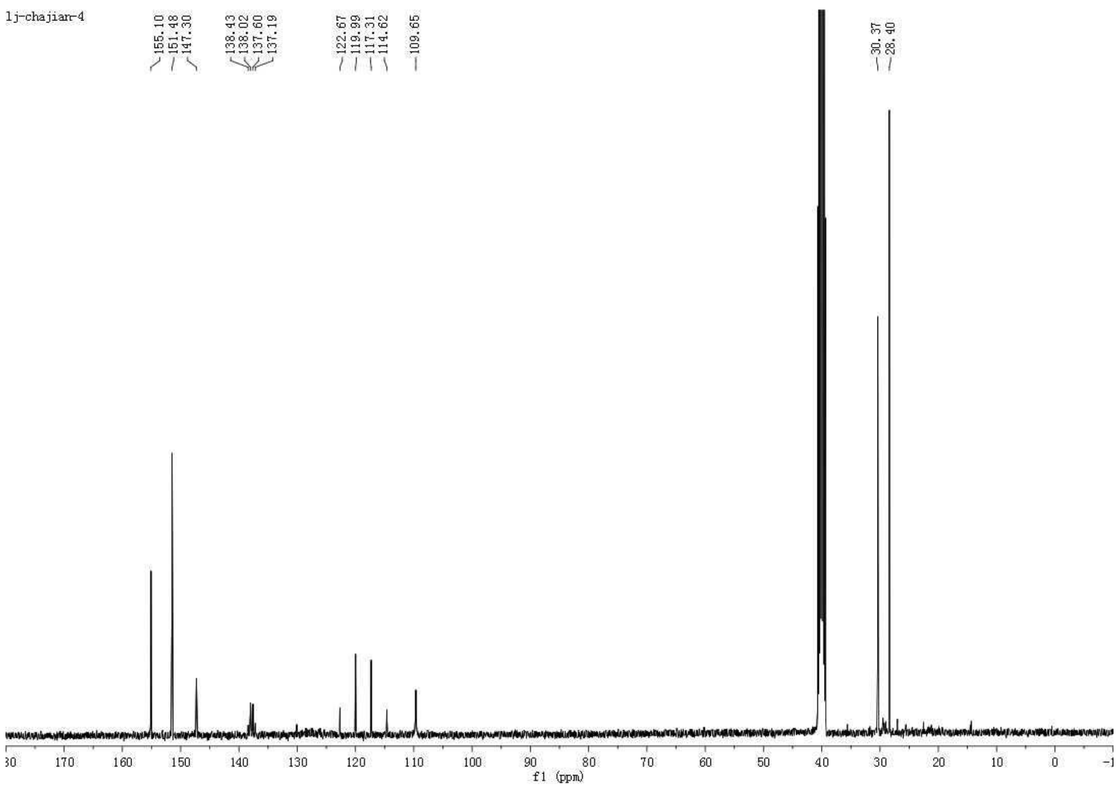

Supplementary Figure 33. ^{13}C NMR spectrum of 1,3-dimethyl-8-(trifluoromethyl)-1*H*-purine-2,6(3*H*,7*H*)-dione (**2t**)

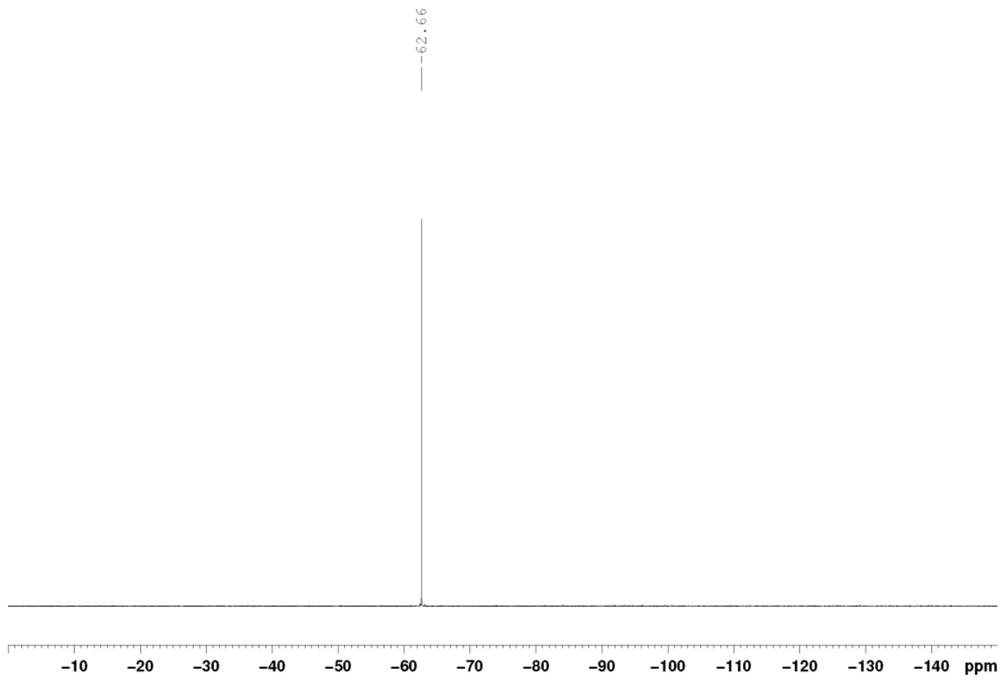

Supplementary Figure 34. ^{19}F NMR spectrum of 1,3-dimethyl-8-(trifluoromethyl)-1*H*-purine-2,6(3*H*,7*H*)-dione (**2t**)

3,7-dimethyl-1-(5-oxohexyl)-8-(trifluoromethyl)-1*H*-purine-2,6(3*H*,7*H*)-dione (**2u**)

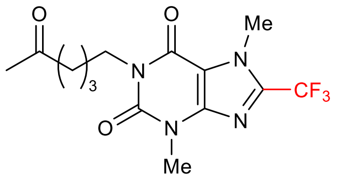

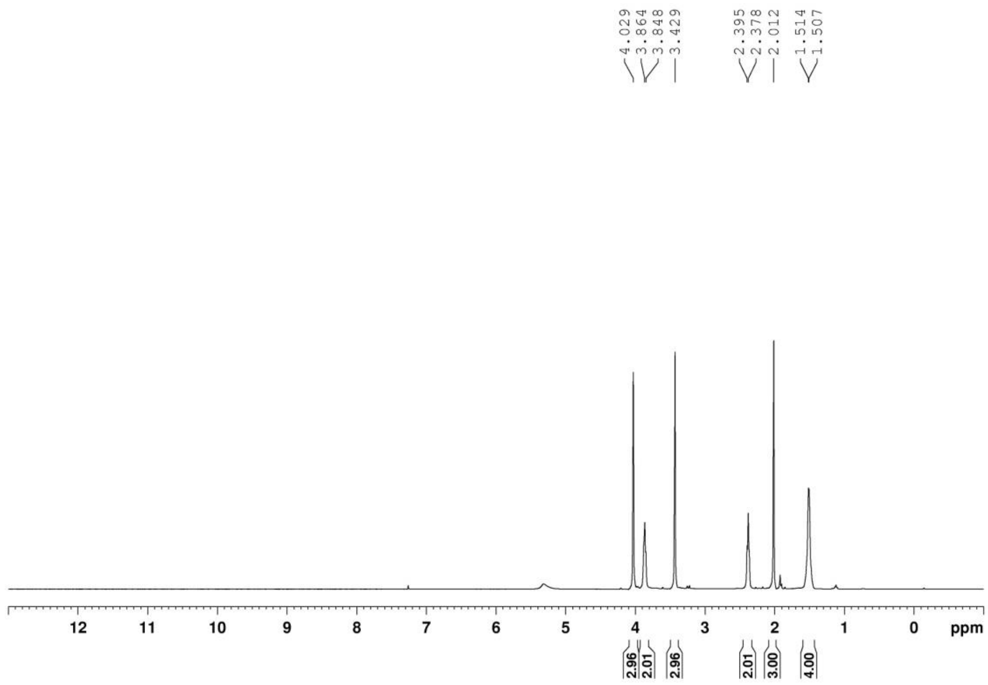

Supplementary Figure 35. ^{1}H NMR spectrum of 3,7-dimethyl-1-(5-oxohexyl)-8-(trifluoromethyl)-1*H*-purine-2,6(3*H*,7*H*)-dione (**2u**)

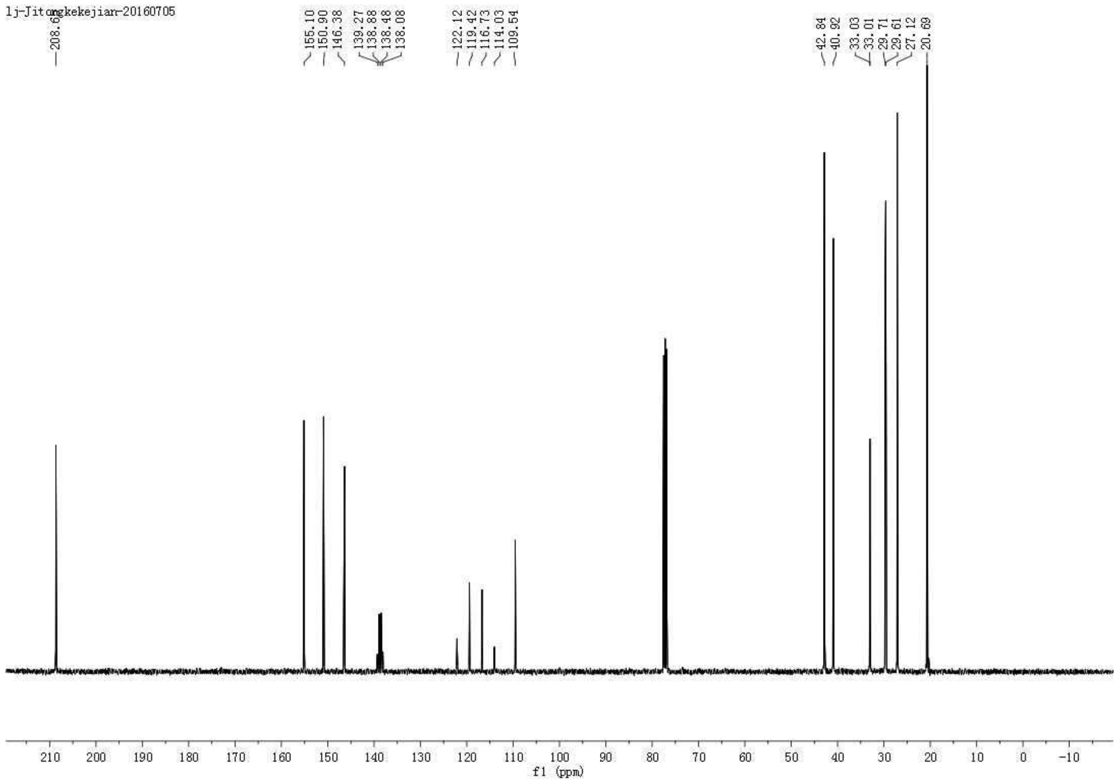

Supplementary Figure 36. ^{13}C NMR spectrum of 3,7-dimethyl-1-(5-oxohexyl)-8-(trifluoromethyl)-1*H*-purine-2,6(3*H*,7*H*)-dione (**2u**)

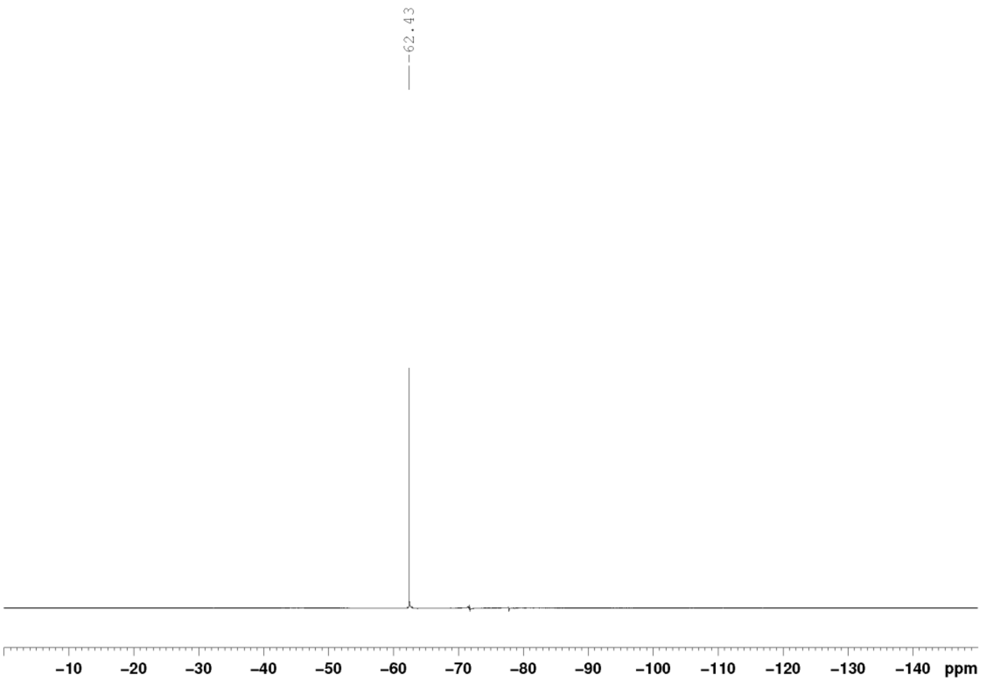

Supplementary Figure 37. ^{19}F NMR spectrum of 3,7-dimethyl-1-(5-oxohexyl)-8-(trifluoromethyl)-1*H*-purine-2,6(3*H*,7*H*)-dione (**2u**)

3,7-dimethyl-8-(trifluoromethyl)-1*H*-purine-2,6(3*H*,7*H*)-dione (**2v**)

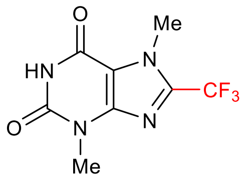

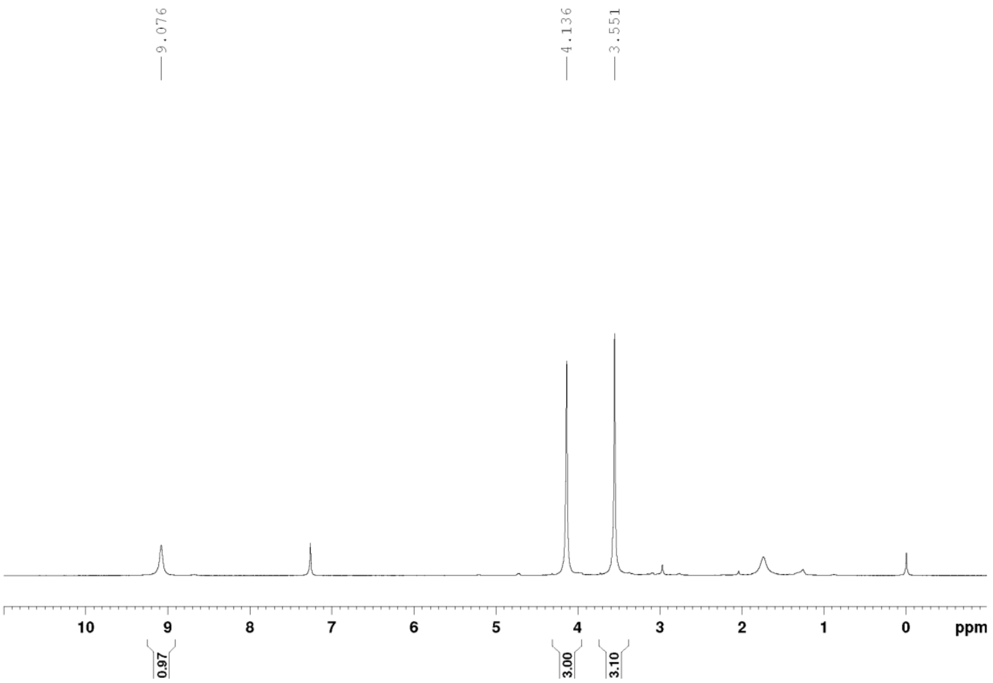

Supplementary Figure 38. ^{1}H NMR spectrum of 3,7-dimethyl-8-(trifluoromethyl)-1*H*-purine-2,6(3*H*,7*H*)-dione (**2v**)

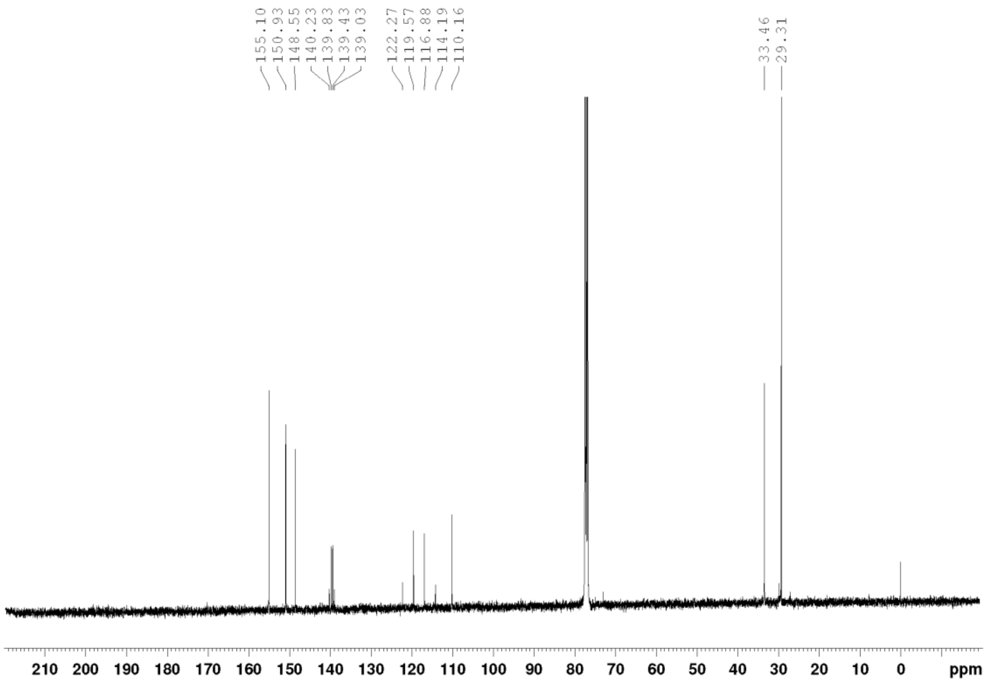

Supplementary Figure 39. ^{13}C NMR spectrum of 3,7-dimethyl-8-(trifluoromethyl)-1*H*-purine-2,6(3*H*,7*H*)-dione (**2v**)

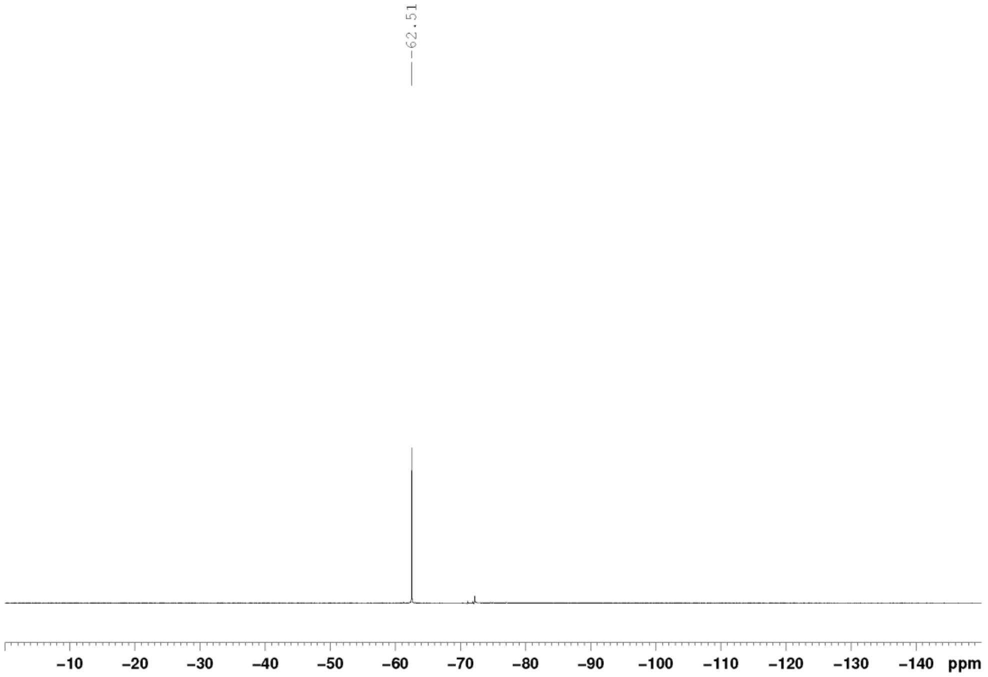

Supplementary Figure 40. ^{19}F NMR spectrum of 3,7-dimethyl-8-(trifluoromethyl)-1*H*-purine-2,6(3*H*,7*H*)-dione (**2v**)

3-(trifluoromethyl)-1*H*-pyrazolo[3,4-*d*]pyrimidin-4(2*H*)-one (**2w**)

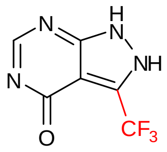

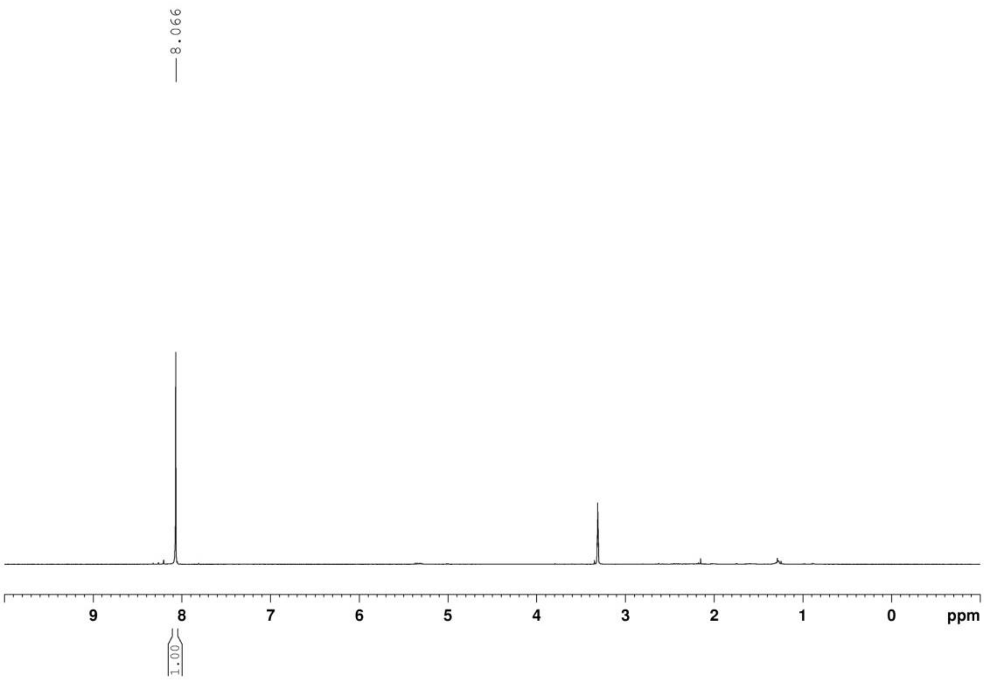

Supplementary Figure 41. ^{1}H NMR spectrum of 3-(trifluoromethyl)-1*H*-pyrazolo[3,4-*d*]pyrimidin-4(2*H*)-one (**2w**)

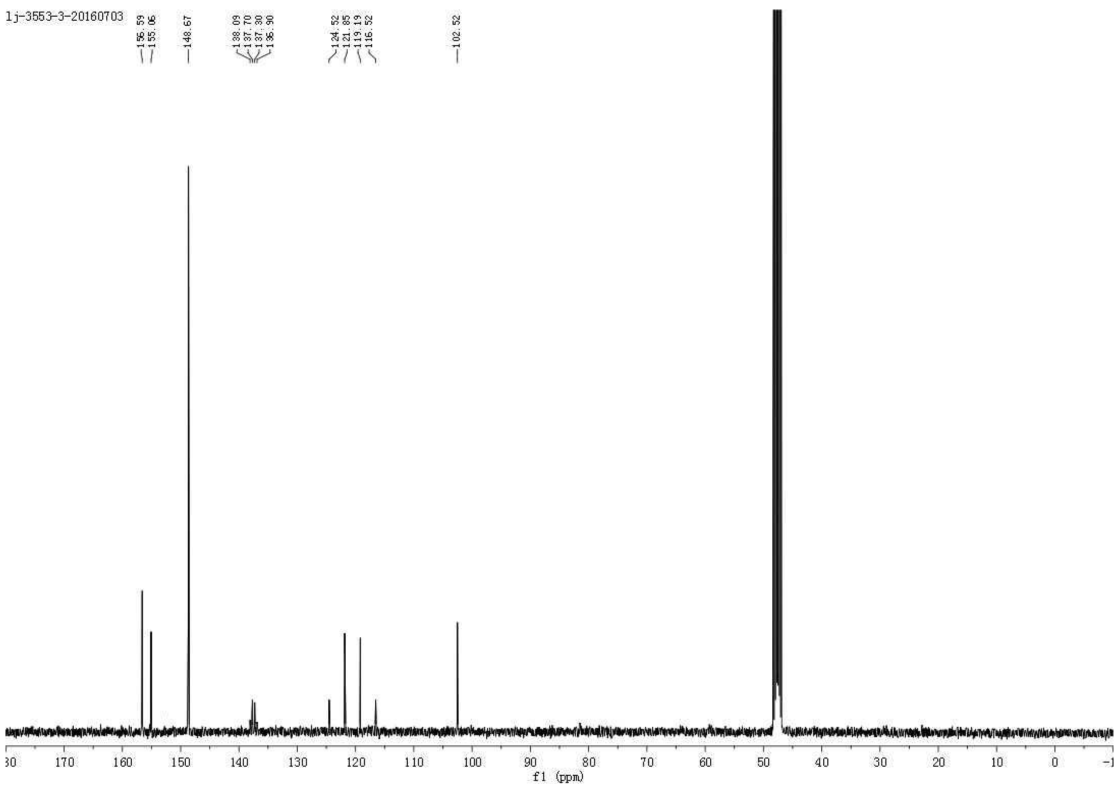

Supplementary Figure 42. ^{13}C NMR spectrum of 3-(trifluoromethyl)-1*H*-pyrazolo[3,4-*d*]pyrimidin-4(2*H*)-one (**2w**)

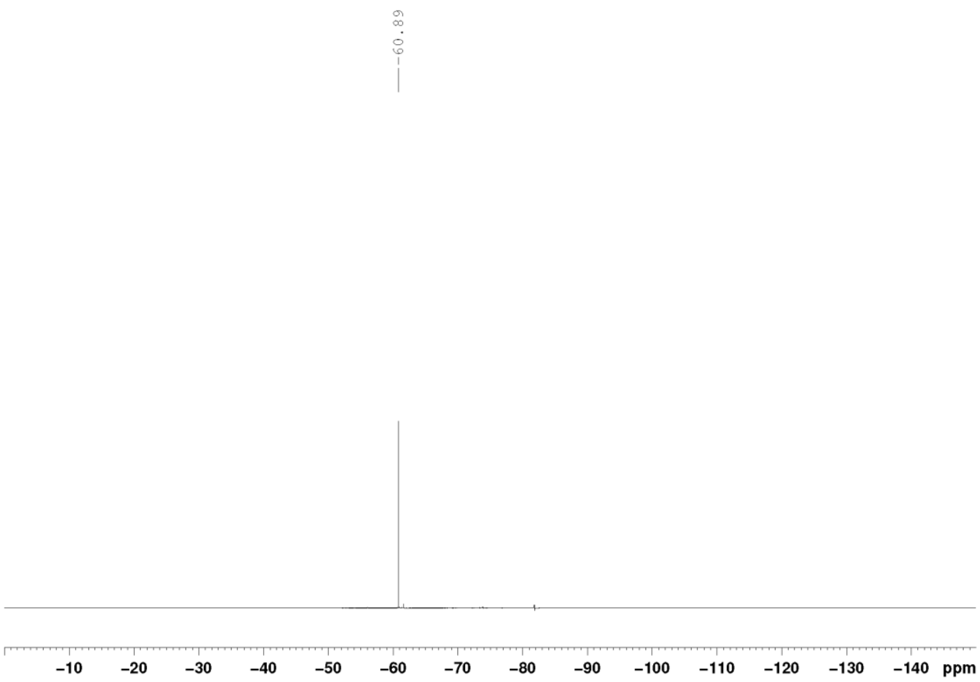

Supplementary Figure 43. ^{19}F NMR spectrum of 3-(trifluoromethyl)-1*H*-pyrazolo[3,4-*d*]pyrimidin-4(2*H*)-one (**2w**)

2-(trifluoromethyl)phenyl acetate (*o*-CF_3-**2k**) 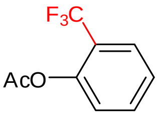

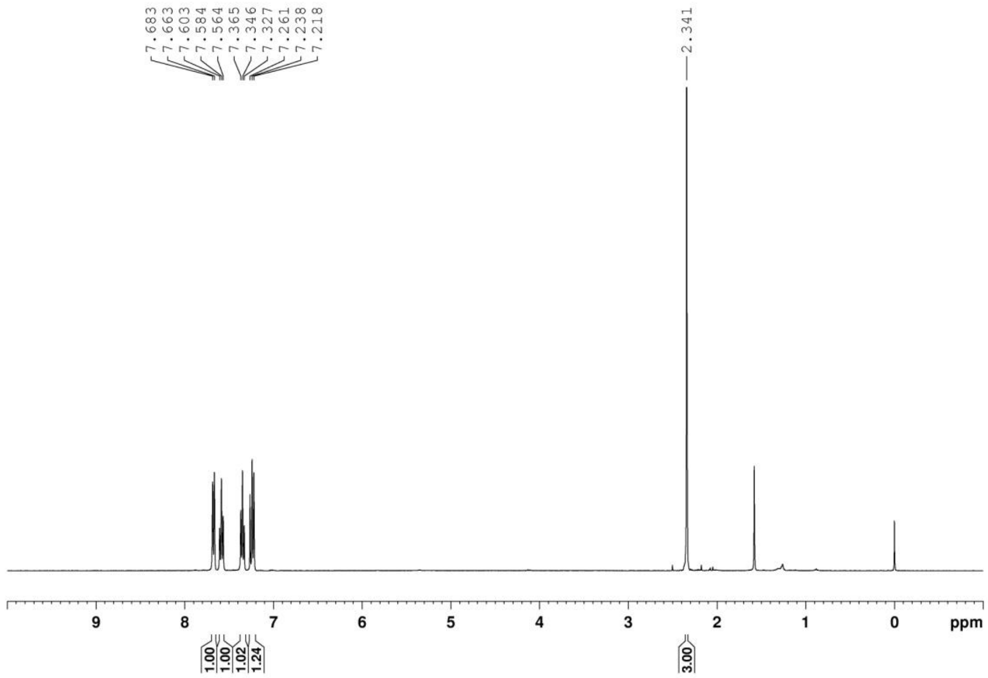

Supplementary Figure 44. ^{1}H NMR spectrum of 2-(trifluoromethyl)phenyl acetate (*o*-CF_3-**2k**)

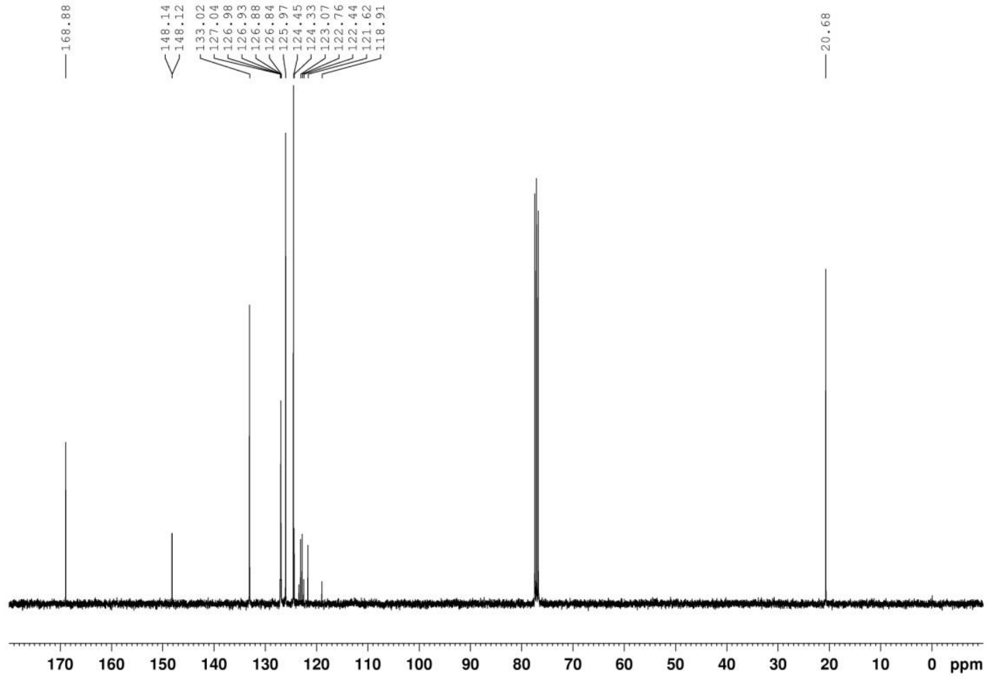

Supplementary Figure 45. ^{13}C NMR spectrum of 2-(trifluoromethyl)phenyl acetate (*o*-CF_3-**2k**)

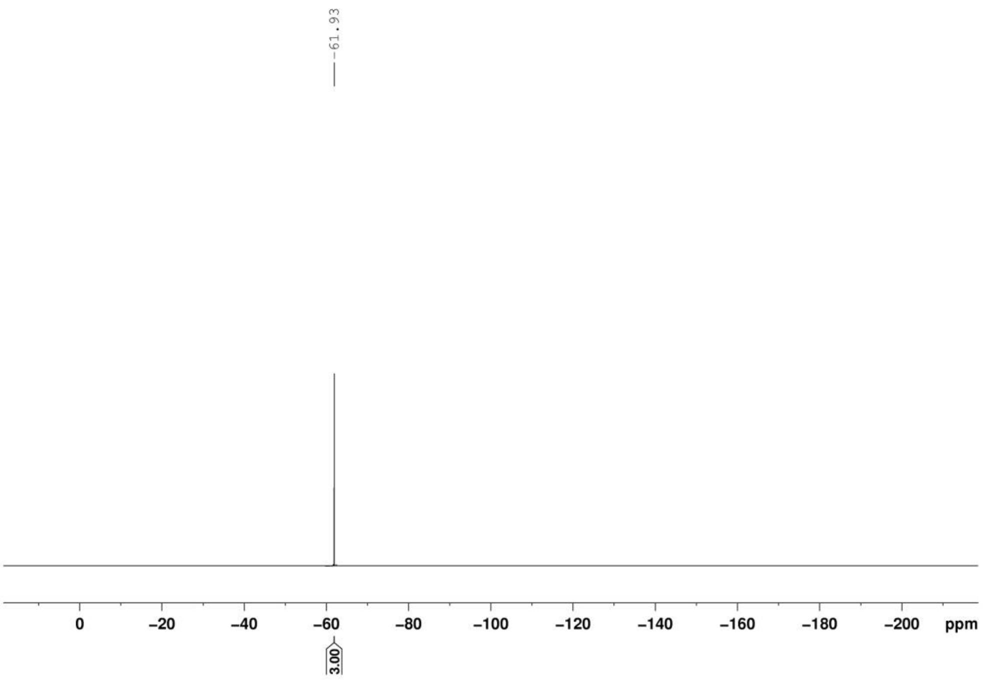

Supplementary Figure 46. ^{19}F NMR spectrum of 2-(trifluoromethyl)phenyl acetate (*o*-CF_3-**2k**)

3-(trifluoromethyl)phenyl acetate (*m*-CF_3-**2k**)

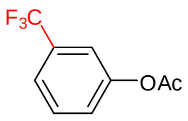

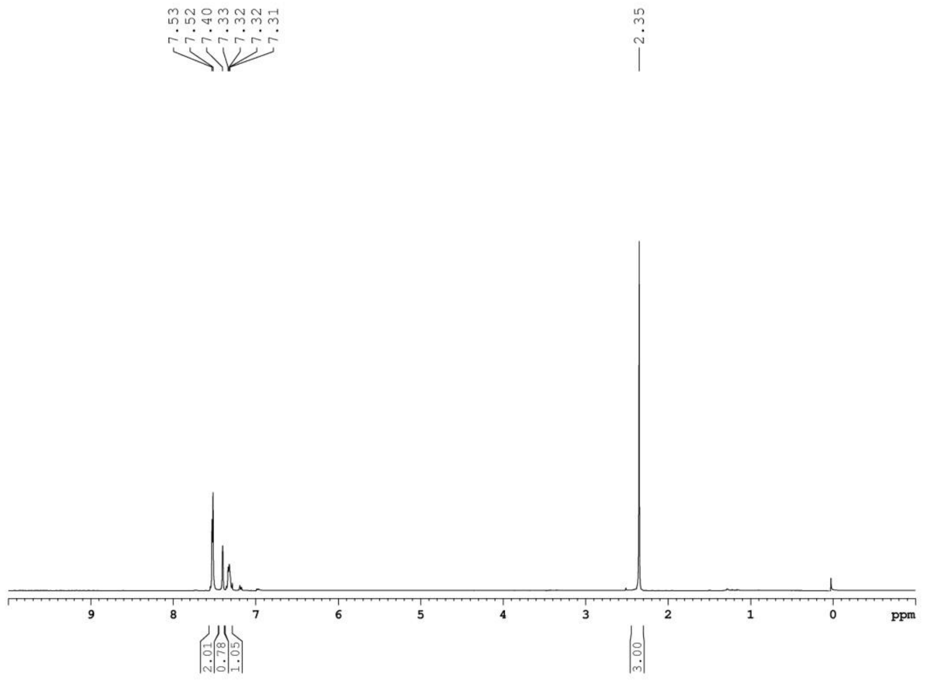

Supplementary Figure 47. ^{1}H NMR spectrum of 3-(trifluoromethyl)phenyl acetate (*m*-CF_3-**2k**)

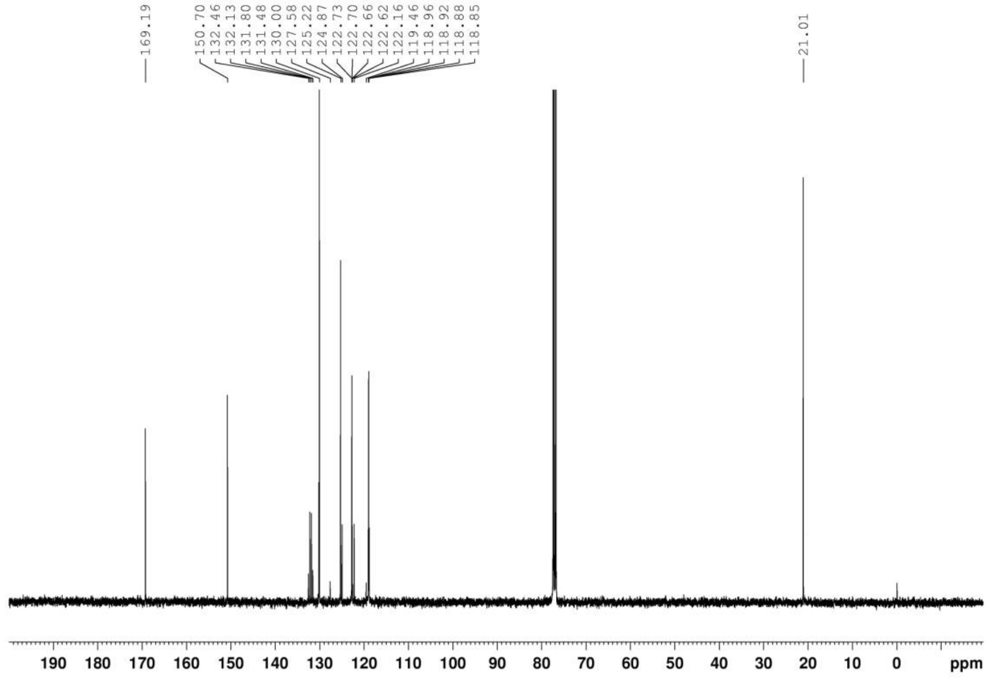

Supplementary Figure 48. ^{13}C NMR spectrum of 3-(trifluoromethyl)phenyl acetate (*m*-CF_3-**2k**)

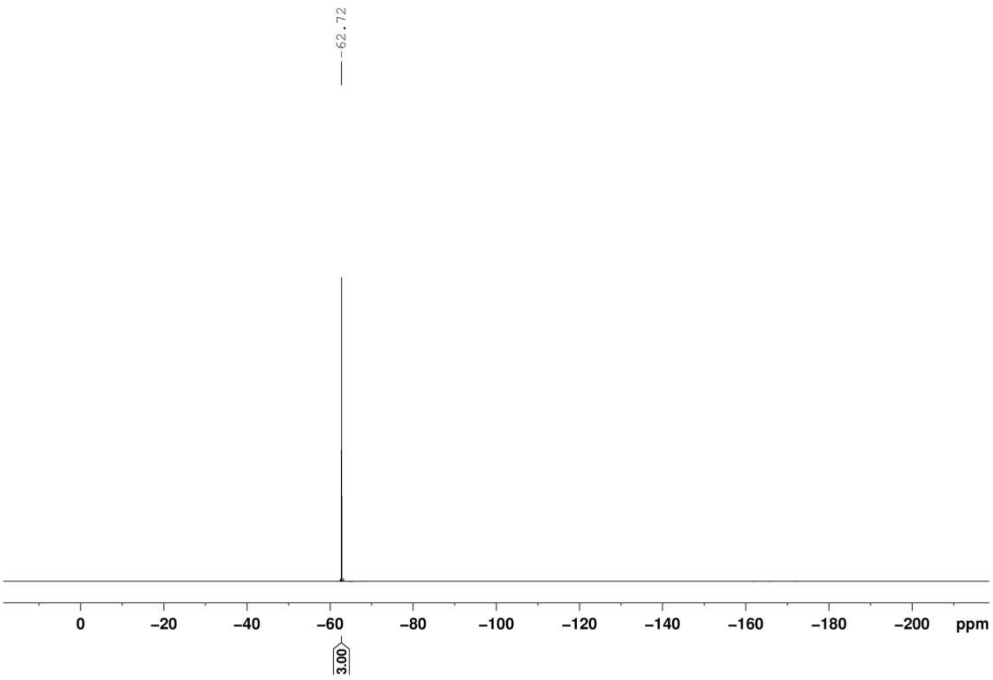

Supplementary Figure 49. ^{19}F NMR spectrum of 3-(trifluoromethyl)phenyl acetate (*m*-CF_3-**2k**)

4-(trifluoromethyl)phenyl acetate (p-CF_3-**2m**) 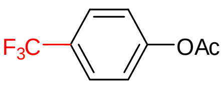

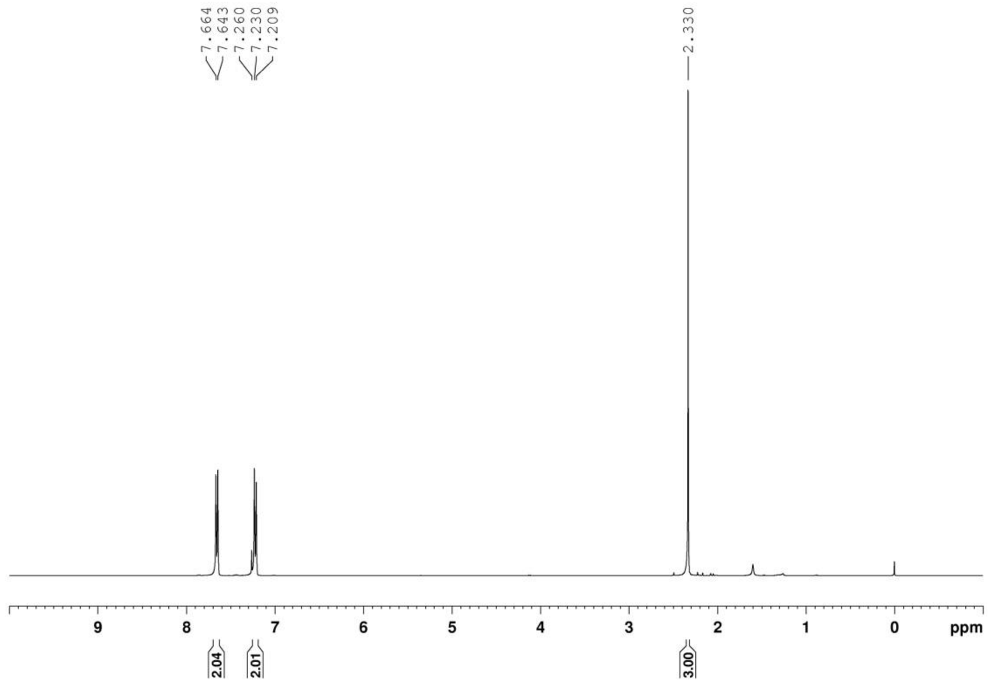

Supplementary Figure 50. ^{1}H NMR spectrum of 4-(trifluoromethyl)phenyl acetate (p-CF_3-**2k**)

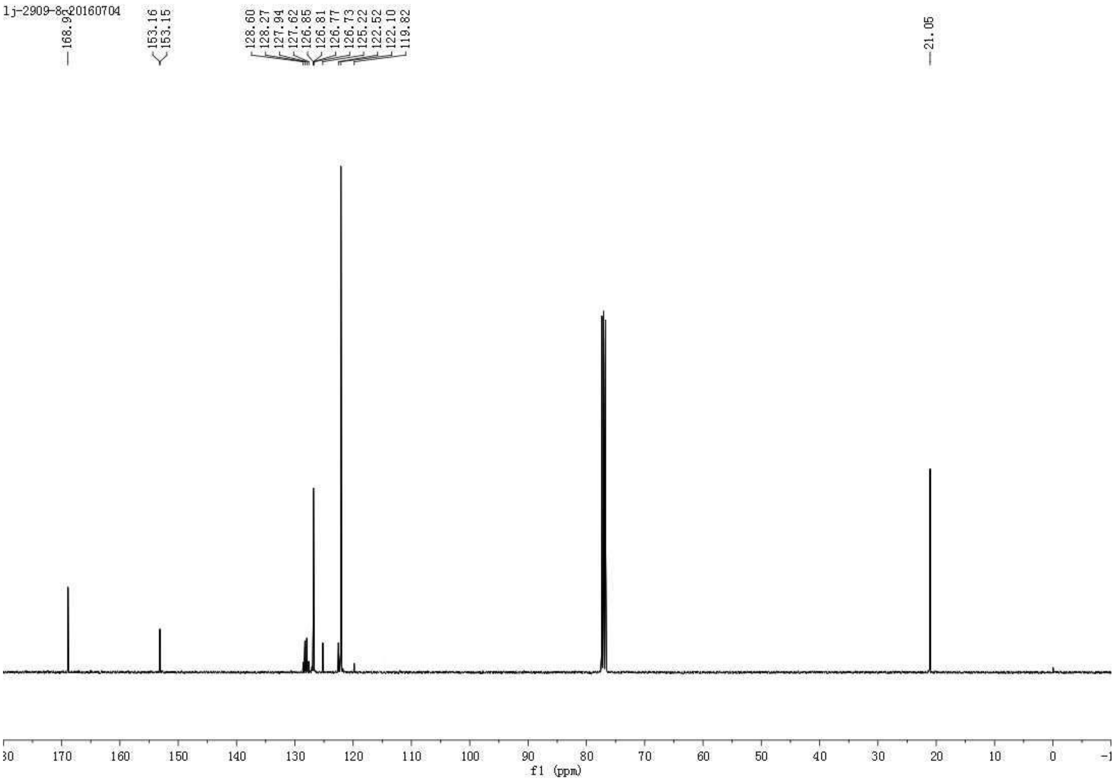

Supplementary Figure 51. ^{13}C NMR spectrum of 4-(trifluoromethyl)phenyl acetate (p-CF_3-**2k**)

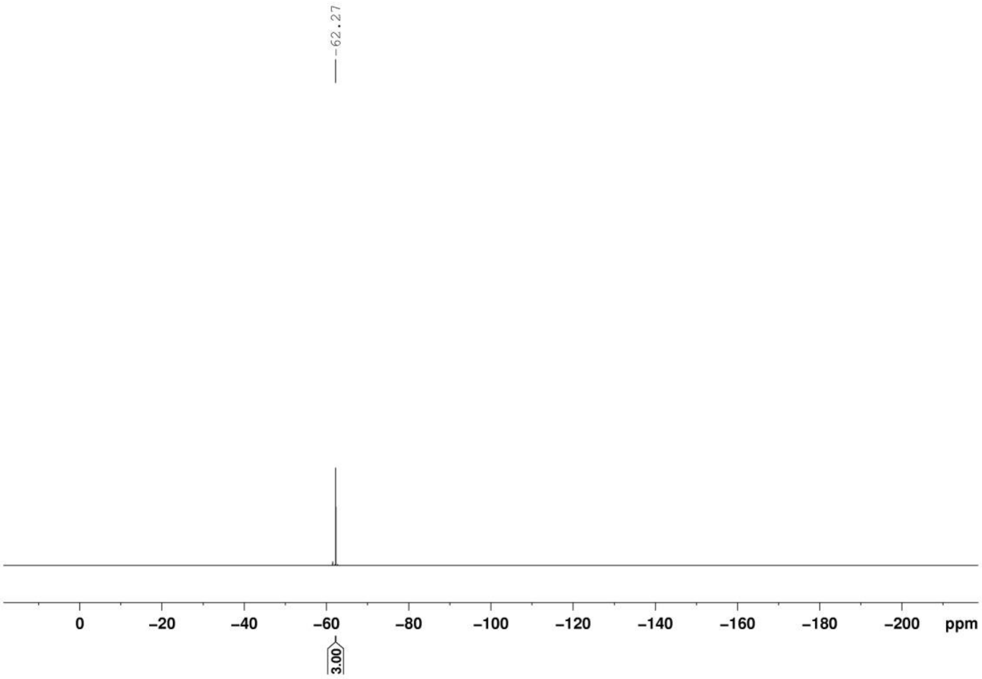

Supplementary Figure 52. ^{19}F NMR spectrum of 4-(trifluoromethyl)phenyl acetate (*p*-CF_3-**2k**)

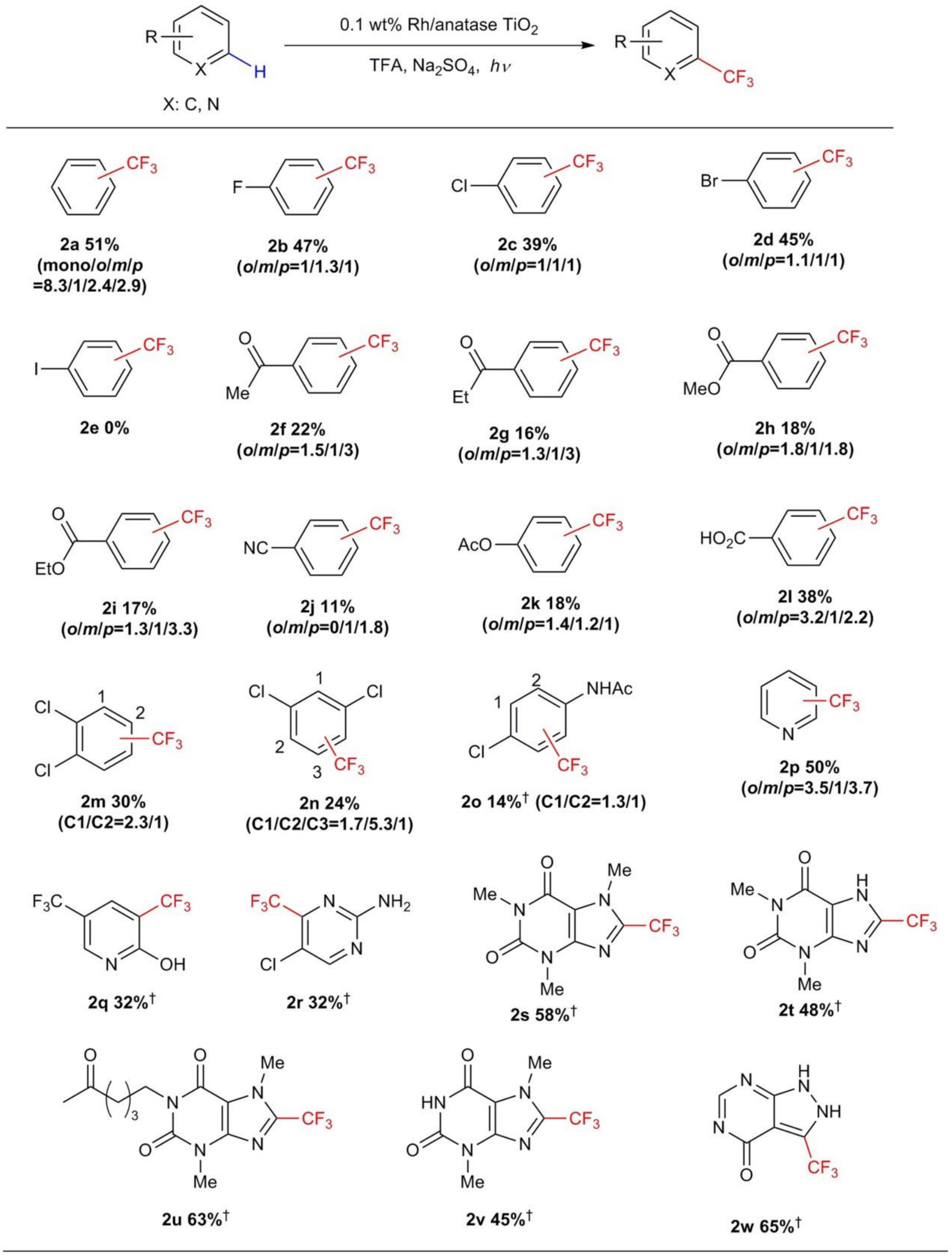

Supplementary Figure 53. photo-driven C-H trifluoromethylation of substituted benzenes and nitrogen-containing heteroarenes using $NaSO_4$ in place of NaS_2O_8. Reaction conditions: substrates (0.5 mmol), 0.1 wt% Rh/anatase TiO_2 NPs (20-40 mol%), Na_2SO_4 (1 equiv.), TFA (10-15 mL), 365 nm UV, room temperature, 24-48 h. †Isolated yield.

Supplementary Table 1. Selected screening results of photo-induced trifluoromethylation of caffeine[a]

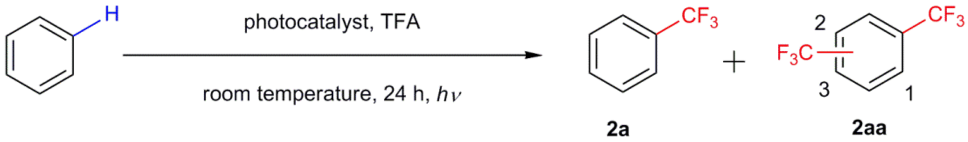

entry	Variation from the standard condition	yield[b]
1	CF_3CO_2Li (1 equiv.) in TFA	trace
2	CF_3CO_2K (1 equiv.) in TFA	trace
3	TFA (1 mL) in DMF (14 mL) instead of TFA	n.r.
4	TFA (1 mL) in DME (14 mL) instead of TFA	n.r.
5	TFA (1 mL) in MeCN (14 mL) instead of TFA	n.r.
6	TFA (1 mL) in DMSO (14 mL) instead of TFA	n.r.
7	TFA (1 mL) in Toluene (14 mL) instead of TFA	n.r.
8	TFA (1 mL) in MeOH (14 mL) instead of TFA	n.r.
9	TFA (1 mL) in NMP (14 mL) instead of TFA	n.r.
10	TFA (1 mL) in $CHCl_3$ (14 mL) instead of TFA	n.r.
11	TFA (1 mL) in CH_2Cl_2 (14 mL) instead of TFA	n.r.
12	TFA (1 mL) in H_2O (14 mL) instead of TFA	n.r.
13	TFA (1 mL) in CCl_4 (14 mL) instead of TFA	n.r.
14	TFA (1 mL) in Nitrobenzene (14 mL) instead of TFA	n.r.
15	TFA (1 mL) in DCE (14 mL) instead of TFA	n.r.
16	TFA (1 mL) in benzonitrile (14 mL) instead of TFA	n.r.
17	TFA (1 mL) in pentafluorobenzene (14 mL) instead of TFA	n.r.
18	TFA (1 mL) in $CNCH_2CO_2Et$ (14 mL) instead of TFA	3
19	TFA (1 mL) in $CNCH_2CO_2Me$ (14 mL) instead of TFA	3
20	TFA (1 mL) in pentafluorobenzonitrile (14 mL) instead of TFA	5
21	$NaNO_2$ (1 equiv.) as additive	0
22	$Ca(ClO)_2$ (1 equiv.) as additive	0
23	$2KHSO_5$•$KHSO_4$•K_2SO_4 (1 equiv.) as additive	0
24	$K_2S_2O_8$ (1 equiv.) as additive	39
25	$NaBrO_5$ (1 equiv.) as additive	8
26	MnO_2 (1 equiv.) as additive	40
27	$KMnO_4$ (1 equiv.) as additive	28
28	$Ce(NO_3)_3$ (1 equiv.) as additive	0
29	$Ce(NH_4)_2(NO_3)_3$ (1 equiv.) as additive	0
30	$Ce(SO_4)_2$ (1 equiv.) as additive	0
31	$Ce(OAc)_3$ (1 equiv.) as additive	10
32	*m*-CPBA (1 equiv.) as additive	6
33	^{t}BuOOH (1 equiv.) as additive	7
34	^{t}BuOO^{t}Bu (1 equiv.) as additive	6
35	$PhCO_3$^{t}Bu (1 equiv.) as additive	0

36	DDQ (1 equiv.) as additive	10
37	$P_2W_{24}O_{77}$ (1 equiv.) as additive	6
38	Na_2WO_4 (1 equiv.) as additive	0
39	K_2WO_4 (1 equiv.) as additive	0
40	AgTFA (2 equiv.) as additive	38
41	AgOTf (2 equiv.) as additive	26
42	AgOTs (2 equiv.) as additive	7
43	Ag_2CO_3 (2 equiv.) as additive	29
44	$AgBF_4$ (2 equiv.) as additive	12
45	$AgPF_6$ (2 equiv.) as additive	14
46	Ag_2O (2 equiv.) as additive	40
47	AgF (2 equiv.) as additive	24
48	$AgNO_3$ (2 equiv.) as additive	0
49	AgO (2 equiv.) as additive	23
50	Ag_3PO_4 (2 equiv.) as additive	46
51	AgOAc (2 equiv.) as additive	31
52	K_2SO_4 (2 equiv.) as additive	30
53	$(NH_4)_2SO_4$ (2 equiv.) as additive	26
54	$ZnSO_4$ (2 equiv.) as additive	11
55	$MgSO_4$ (2 equiv.) as additive	10
56	$CaSO_4$ (2 equiv.) as additive	11
57	$NaHSO_4$ (2 equiv.) as additive	10
58	$KHSO_4$ (2 equiv.) as additive	8
59	Li_2SO_4 (2 equiv.) as additive	29
60	Cs_2SO_4 (2 equiv.) as additive	31
61	$Al_2(SO_4)_3$ (2 equiv.) as additive	0
62	$H_2SO_4 \bullet 0.5\ SO_3$ (1 mL) as additive	33
63	26W 365 nm UV instead of 250W 365 nm UV, $Na_2S_2O_8$ (20 mol%) as additive	trace
64	100W 365 nm UV instead of 250W 365 nm UV, $Na_2S_2O_8$ (20 mol%) as additive	39
65	400W 365 nm UV instead of 250W 365 nm UV, $Na_2S_2O_8$ (20 mol%) as additive	48
66	1kW 365 nm UV instead of 250W 365 nm UV, $Na_2S_2O_8$ (20 mol%) as additive	trace
67	24W visible light instead of 250W 365 nm UV, $Na_2S_2O_8$ (20 mol%) as additive	n.r.
68	46W visible light instead of 250W 365 nm UV, $Na_2S_2O_8$ (20 mol%) as additive	n.r.
69	300W visible light instead of 250W 365 nm UV, $Na_2S_2O_8$ (20 mol%) as additive	trace
70	Cu/anatase TiO_2 instead of 0.1wt% Rh/anatase TiO_2	35
71	Au/anatase TiO_2 instead of 0.1wt% Rh/anatase TiO_2	39

72	Pt/anatase TiO_2 instead of 0.1wt% Rh/anatase TiO_2	35
73	Pd/anatase TiO_2 instead of 0.1wt% Rh/anatase TiO_2	36
74	Ag/anatase TiO_2 instead of 0.1wt% Rh/anatase TiO_2	43

[a] standad conditions: benzene (0.5 mmol), 0.1wt% Rh/anatase TiO_2 as photocatalytst (20 mol%), distilled TFA (15 mL), room temperature, 365 nm irradiation(250 W), 24h.

[b] yield determined by ^{19}F NMR using 1-methoxy-4-(trifluoromethoxy)benzene as the internal standard. The yield includes benzotrifluoride (**2a**) and bi-trifluoromethyl-substituted benzene (**3a**)

Supplementary Methods

General Information. All reagents for catalyst preparations and photo-induced trifluoromethylation experiments, including all substrates and some products, were purchased from commercial sources without further purification unless otherwise noted. Solvents including trifluoroacetic acid (TFA), acetonitrile (MeCN), N,N-dimethylformamide (DMF), dimethoxyethane (DME), dimethyl sulphoxide (DMSO), Toluene, methanol, N-methyl-2-pyrrolidone (NMP), $CHCl_3$, CH_2Cl_2, CCl_4, nitrobenzene, dichloroethane (DCE), benzonitrile were used after distillation for purification.

Instrument. The 365 nm ultra-violet irradiation was obtained by using high pressure mercury lamp (100 W/250 W/400 W/1 kW). The visible light irradiation was provided by 300 W Xe lamp (PLS-SXE300C, Beijing Perfect Light Co.) equipped with a 420 nm cutoff filter. XPS analysis was carried out on a Thermofisher ESCALAB 250 X-ray photoelectron spectrometer with a chromatized Al Kα source (15 kV, 150W). XRD measurements were carried out on Rigaku MiniFlexⅡ using Cu Kα as radiation source (λ = 0.15064 nm) at 30 kV and 15 mA. TEM and SEAD observation was made on JEOL JEM-2010. The preparations of TEM samples were carried out by depositing a drop of the nanoparticle suspension, which was redispersed by ultrasonics, onto a continuous carbon-coated copper grid and dried at room temperature under atmospheric pressure. Gas chromatographic (GC) analysis for H_2 determination was conducted using an Agilent 7820A gas chromatography equipped with a thermal conductivity detector (TCD) and a TD-01 packed column, using Ar as the carrier gas. ^{1}H, ^{13}C, and ^{19}F NMR spectra were recorded on Burker Avance 400 NMR spectrometer. Data for ^{1}H NMR are recorded as follows: chemical shift (δ, ppm), multiplicity (s, singlet; d, doublet; t, triplet; q, quartet; m, multiplet), coupling constant (Hz), integration. Data for ^{13}C NMR are reported in terms of chemical shift (δ, ppm). ^{19}F NMR spectra are reported in terms of chemical shift (δ, ppm).

Preparation of 0.1 wt% Rh-modified anatase TiO_2 nanopaticles. In a 30 mL quartz round bottom flask, a mixture of 60 nm anatase TiO_2 (3.0 g) and $RhCl_3$•xH_2O (0.023 g, Rh content of 39 %) was deoxygenated and filled with N_2, followed by addition of deoxygenated methanol (30 mL). The suspension was stirred with an irradiation of 250 W high pressure Hg lamp (365 nm UV) for 48 h. After illumination, nanocatalysts were separated from the solution by centrifugation (8000 rpm, 2 min, 298 K). The separated nanocatalysts were washed for three times by ethanol and three times by water, dried in vacuum at 298 K for 8 h. The Rh content of as-prepared nanocatalyst was 0.1 wt% by the inductively coupled plasma (ICP) analysis.

Preparation of anatase TiO_2 nanopaticles modified by other transition metals (Pd, Au, Ag, Pt, Cu). The anatase TiO_2 nanocatalysts modified by other transition metals were prepared by following the method for preparation of 0.1 wt% Rh-modified TiO_2 nanocatalyst. In these processes, $PdCl_2$ (0.0151 g), H_3AuCl_6 solution in methanol (0.94 mL, 0.02g H_3AuCl_6 /mL), $CuCl_2$ (0.0191 g), $AgNO_3$ (0.0143 g) and H_2PtCl_6•$6H_2O$ (0.024 g) were used, respectively, with 60 nm

anatase TiO_2 (3.0g) and methanol (30 mL).

Typical reaction A (trifluoromethylation reaction of caffeine was used as an example). photocatalyst (0.016 g, 0.2 mmol), caffeine (0.0971 g, 0.5 mmol) and $Na_2S_2O_8$ (0.0238 g, 0.1 mmol) were introduced into a Schlenk tube. Then, the tube was fitted with a rubber septum. After evacuation and N_2 backfill for three times, distilled trifluoroacetic acid (15 mL) was added to the Schlenk tube through the rubber septum using syringes, and the rubber septum was replaced by a Teflon cap under N_2 flow. The reaction was performed under illumination of 250 W high pressure Hg lamp (365 nm UV) at room temperature for 48 hours. After reaction, the trifluoroacetic acid was removed under reduced pressure and the residue was purified by flash chromatography on silica gel (eluent: EtOAc/hexanes) to provide the corresponding product. The yield were also determined by ^{19}F NMR spectrum using 1-methoxy-4-(trifluoromethoxy)benzene (76 μL, 0.5 mmol, δ -58.4 ppm) as an internal reference.

Typical reaction B (trifluoromethylation reaction of caffeine was used as an example). Typical reaction B is identical to typical reaction A except that Na_2SO_4 was used instead of $Na_2S_2O_8$.

Typical gram scale reaction (trifluoromethylation reaction of caffeine was used as an example). 0.1 wt% Rh/anatase TiO_2 nanocatalysts of (0.04 g, 0.5 mmol), caffeine (1 g, 5 mmol) and $Na_2S_2O_8$ (0.119 g, 0.5 mmol) were introduced into a Schlenk tube. Then, the tube was fitted with a rubber septum. After evacuation and N_2 backfill for three times, distilled trifluoroacetic acid (60 mL) was added to the Schlenk tube through the rubber septum using syringes, and the rubber septum was replaced by a Teflon cap under N_2 flow. The reaction was performed under illumination of 250 W high pressure Hg lamp (365 nm UV) at room temperature for 120 hours. After reaction, the trifluoroacetic acid was removed under reduced pressure and the residue was purified by flash chromatography on silica gel (eluent: EtOAc/hexanes) to provide the corresponding product.

Radical trapping experiment with TEMPO. 0.1 wt% Rh/anatase TiO_2 nanocatalysts (0.008 g, 0.1 mmol), TEMPO (0.0781 g, 0.5 mmol) and Na_2SO_4 (0.14 g, 1 mmol), were introduced into a Schlenk tube. Then, the tube was fitted with a rubber septum. After evacuation and N_2 backfill for three times, benzene (46 μL, 0.5 mmol) and 2 mL distilled trifluoroacetic acid was added to the Schlenk tube through the rubber septum using syringes, and the rubber septum was replaced by a Teflon cap under N_2 flow. The reaction was performed under illumination of 100 W high pressure Hg lamp (365 nm UV) at room temperature for 2 hours. The resultant mixture was analyzed by ^{19}F NMR spectroscopy using 1-methoxy-4-(trifluoromethoxy)benzene (76 μL, 0.5 mmol, δ -58.4 ppm) as the internal standard.

Detection of H_2 and CO_2 formed in the control experiment of trifluoroacetic acid. 0.1 wt% Rh/anatase TiO_2 nanocatalysts (0.016 g, 0.2 mmol) and Na_2SO_4 (0.14 g, 1 mmol) were introduced into a Schlenk tube. Then, the tube was fitted with a rubber septum. After evacuation and N_2 backfill for three times, distilled trifluoroacetic acid (15 mL) was added to the Schlenk tube through the rubber septum using syringes, and the rubber septum was replaced by a Teflon cap

under N_2 flow. The reaction was performed under illumination of 250 W high pressure Hg lamp (365 nm UV) at room temperature for 24 hours. After reaction, the atmosphere of reaction (1 mL) in schlenk tube was injected into GC instrument. The oven temperature was held constant at 40 °C for 20 min, then it was raised to 250 °C with 15°C/min. Inlet and detector temperature were set at 120 °C and 200 °C, respectively.

Synthesis of CF_3I. 0.1 wt% Rh/anatase TiO_2 nanocatalysts (0.016 g, 0.1 mmol) and I_2 (0.127 g, 1 mmol) were introduced to a Schlenk tube. Then, the tube was fitted with a rubber septum. After evacuation and N_2 backfill for three times, distilled trifluoroacetic acid (15 mL) was added to the Schlenk tube through the rubber septum using syringes, and the rubber septum was replaced by a Teflon cap under N_2 flow. The reaction was performed under illumination of 250 W high pressure Hg lamp (365 nm UV) at room temperature for 24 hours. The yield was determined by ^{19}F NMR spectrum using 1-methoxy-4-(trifluoromethoxy)benzene (76 μL, 0.5 mmol, δ -58.4 ppm) as the internal standard..

Characterization of photocatalysts. The characterization of as-prepared photocatalysts was carried out using XPS, XRD, TEM, HRTEM, and ICP.
After catalytic reaction, the suspension was centrifuged (8000 rpm, 2 min, 298 K) for separating nanocatalysts from the solution. And then the separated nanocatalysts were washed for 3 times with ethanol and 3 times with water, dried in vacuum at 298 k for 10 h. The measurements of separated nanocatalysts for XPS, XRD, TEM and ICP were performed.

Substrates:

2a 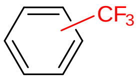

The typical reaction A was followed with benzene (46 μL, 0.5 mmol), 0.1wt% Rh/anatase TiO_2 (0.008 g, 0.1 mmol), $Na_2S_2O_8$ (0.0238 g, 0.1 mmol) and a reaction time of 24 hours. The reaction mixture was analyzed directly by ^{19}F NMR (70 %, monosubstituted/*o*/*m*/*p* = 17.3/2.8/1/1.8).
The typical reaction B was followed with benzene (46 μL, 0.5 mmol), 0.1wt% Rh/anatase TiO_2 (0.008 g, 0.1 mmol), Na_2SO_4 (0.14 g, 1 mmol) and a reaction time of 24 hours. The reaction mixture was analyzed directly by ^{19}F NMR (51 %, monosubstituted/*o*/*m*/*p* = 8.3/1/2.4/2.9).
Due to the availability of the products, no purification was attempted on this reaction mixture. The fluorine signals of the products were identical to those of the commercial samples.

2b 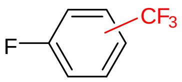

The typical reaction A was followed with fluorobenzene (47 μL, 0.5 mmol), 0.1wt% Rh/anatase TiO_2 (0.008 g, 0.1 mmol), $Na_2S_2O_8$ (0.0238 g, 0.1 mmol) and a reaction time of 48 hours. The reaction mixture was analyzed directly by ^{19}F NMR (43 %, *o*/*m*/*p* = 1/1.3/2).

The typical reaction B was followed with fluorobenzene (47 μL, 0.5 mmol), 0.1wt% Rh/anatase TiO_2 (0.008 g, 0.1 mmol), Na_2SO_4 (0.14 g, 1 mmol) and a reaction time of 72 hours. The reaction mixture was analyzed directly by ^{19}F NMR (47 %, *o*/*m*/*p* = 1/1.3/1).
Due to the availability of the products, no purification was attempted on this reaction mixture. The fluorine signals of the products were identical to those of the commercial samples.

2c 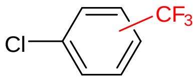

The typical reaction A was followed with chlorobenzene (52 μL, 0.5 mmol), $Na_2S_2O_8$ (0.0238 g, 0.1 mmol), 0.1wt% Rh/anatase TiO_2 (0.008 g, 0.1 mmol) and a reaction time of 48 hours. The reaction mixture was analyzed directly by ^{19}F NMR (65 %, *o*/*m*/*p* = 4.5/3.1/1).
The typical reaction B was followed with chlorobenzene (52 μL, 0.5 mmol), Na_2SO_4 (0.14 g, 1 mmol), 0.1wt% Rh/anatase TiO_2 (0.016 g, 0.2 mmol) and a reaction time of 48 hours. The reaction mixture was analyzed directly by ^{19}F NMR (39 %, *o*/*m*/*p* = 1/1/1).
Due to the availability of the products, no purification was attempted on this reaction mixture. The fluorine signals of the products were identical to those of the commercial samples.

2d 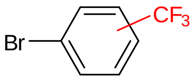

The typical reaction A was followed with bromobenzene (52 μL, 0.5 mmol), $Na_2S_2O_8$ (0.0238 g, 0.1 mmol), 0.1wt% Rh/anatase TiO_2 (0.008 g, 0.1 mmol) and a reaction time of 48 hours. The reaction mixture was analyzed directly by ^{19}F NMR (64 %, *o*/*m*/*p* = 1.2/1/1).
The typical reaction B was followed with bromobenzene (52 μL, 0.5 mmol), Na_2SO_4 (0.14 g, 1 mmol), 0.1wt% Rh/anatase TiO_2 (0.016 g, 0.2 mmol) and a reaction time of 48 hours. The reaction mixture was analyzed directly by ^{19}F NMR (45 %, *o*/*m*/*p* = 1.1/1/1).
Due to the availability of the products, no purification was attempted on this reaction mixture. The fluorine signals of the products were identical to those of the commercial samples.

2e 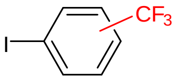

The typical reaction A was followed with iodobenzene (56 μL, 0.5 mmol), $Na_2S_2O_8$ (0.0238 g, 0.1 mmol), 0.1wt% Rh/anatase TiO_2 (0.008 g, 0.1 mmol) and a reaction time of 48 hours. The reaction mixture was analyzed directly by ^{19}F NMR (52 %, *o*/*m*/*p* = 1/1.8/1.8).
Due to the availability of the products, no purification was attempted on this reaction mixture. The fluorine signals of the products were identical to those of the commercial samples.

2f 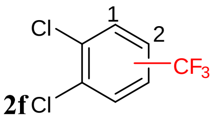

The typical reaction A was followed with 1,2-dichlorobenzene (56 μL, 0.5 mmol), $Na_2S_2O_8$ (0.0238 g, 0.1 mmol), 0.1wt% Rh/anatase TiO_2 (0.016 g, 0.2 mmol) and a reaction time of 24 hours. The reaction mixture was analyzed directly by ^{19}F NMR (63 %, C1/C2 = 1/1.2).
The typical reaction B was followed with 1,2-dichlorobenzene (56 μL, 0.5 mmol), Na_2SO_4 (0.14 g, 1 mmol), 0.1wt% Rh/anatase TiO_2 (0.016 g, 0.2 mmol) and a reaction time of 24 hours. The reaction mixture was analyzed directly by ^{19}F NMR (30 %, C1/C2 = 2.3/1).
Due to the availability of the products, no purification was attempted on this reaction mixture. The fluorine signals of the products were identical to those of the commercial samples.

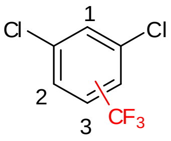

2g

The typical reaction A was followed with 1,3-dichlorobenzene (57 μL, 0.5 mmol), $Na_2S_2O_8$ (0.0238 g, 0.1 mmol), 0.1wt% Rh/anatase TiO_2 (0.016 g, 0.2 mmol) and a reaction time of 24 hours. The reaction mixture was analyzed directly by ^{19}F NMR (54 %, C1/C2/C3 = 5/1.2/1).
The typical reaction A was followed with 1,3-dichlorobenzene (57 μL, 0.5 mmol), Na_2SO_4 (0.14 g, 1 mmol), 0.1wt% Rh/anatase TiO_2 (0.016 g, 0.2 mmol) and a reaction time of 24 hours. The reaction mixture was analyzed directly by ^{19}F NMR (24 %, C1/C2/C3 = 1.7/5.3/1).
Due to the availability of the products, no purification was attempted on this reaction mixture. The fluorine signals of the products were identical to those of the commercial samples.

2h

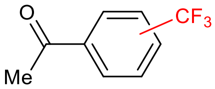

The typical reaction A was followed with acetophenone (58 μL, 0.5 mmol), $Na_2S_2O_8$ (0.0238 g, 0.1 mmol), 0.1wt% Rh/anatase TiO_2 (0.016 g, 0.2 mmol) and reaction time of 48 hours. The reaction mixture was analyzed directly by ^{19}F NMR (46 %, *o*/*m*/*p* = 2.4/1/3).
The typical reaction B was followed with acetophenone (58 μL, 0.5 mmol), Na_2SO_4 (0.14 g, 1 mmol), 0.1wt% Rh/anatase TiO_2 (0.008 g, 0.1 mmol) and reaction time of 48 hours. The reaction mixture was analyzed directly by ^{19}F NMR (22 %, *o*/*m*/*p* = 1.5/1/3).
Due to the availability of the products, no purification was attempted on this reaction mixture. The fluorine signals of the products were identical to those of the commercial samples.

2i

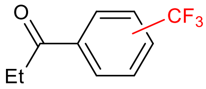

The typical reaction A was followed with propiophenone (66 μL, 0.5 mmol), $Na_2S_2O_8$ (0.0479 g, 0.2 mmol), 0.1wt% Rh/anatase TiO_2 (0.008 g, 0.1 mmol) and a reaction time of 48 hours. The reaction mixture was analyzed directly by ^{19}F NMR (48 %, *o*/*m*/*p* = 3.3/1/4).
The typical reaction B was followed with propiophenone (66 μL, 0.5 mmol), Na_2SO_4 (0.14 g, 1 mmol), 0.1wt% Rh/anatase TiO_2 (0.008 g, 0.1 mmol) and a reaction time of 48 hours. The reaction mixture was analyzed directly by ^{19}F NMR (16 %, *o*/*m*/*p* = 1.3/1/3).
Due to the availability of the products, no purification was attempted on this reaction mixture. The

fluorine signals of the products were identical to those of the commercial samples.

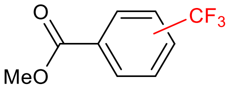

2j

The typical reaction A was followed with methyl benzoate (62 μL, 0.5 mmol), $Na_2S_2O_8$ (0.0479 g, 0.2 mmol), 0.1wt% Rh/anatase TiO_2 (0.008 g, 0.1 mmol) and a reaction time of 48 hours. The reaction mixture was analyzed directly by ^{19}F NMR (57 %, *o*/*m*/*p* = 1.8/1/1.9).
The typical reaction B was followed with methyl benzoate (62 μL, 0.5 mmol), Na_2SO_4 (0.14 g, 1 mmol), 0.1wt% Rh/anatase TiO_2 (0.016 g, 0.2 mmol) and a reaction time of 48 hours. The reaction mixture was analyzed directly by ^{19}F NMR (18 %, *o*/*m*/*p* = 1.8/1/1.8).
Due to the availability of the products, no purification was attempted on this reaction mixture. The fluorine signals of the products were identical to those of the commercial samples.

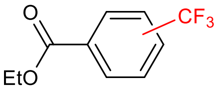

2j

The typical reaction A was followed with ethyl benzoate (72 μL, 0.5 mmol), $Na_2S_2O_8$ (0.0238 g, 0.1 mmol), 0.1wt% Rh/anatase TiO_2 (0.008 g, 0.1 mmol) and a reaction time of 48 hours. The reaction mixture was analyzed directly by ^{19}F NMR (46 %, *o*/*m*/*p* = 1.9/1/1.7).
The typical reaction B was followed with ethyl benzoate (72 μL, 0.5 mmol), Na_2SO_4 (0.14 g, 1 mmol), 0.1wt% Rh/anatase TiO_2 (0.016 g, 0.2 mmol) and a reaction time of 48 hours. The reaction mixture was analyzed directly by ^{19}F NMR (17 %, *o*/*m*/*p* = 1.3/1/3.3).
Due to the availability of the products, no purification was attempted on this reaction mixture. The fluorine signals of the products were identical to those of the commercial samples.

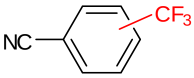

2l

The typical reaction A was followed with benzonitrile (52 μL, 0.5 mmol), $Na_2S_2O_8$ (0.0479 g, 0.2 mmol), 0.1wt% Rh/anatase TiO_2 (0.008 g, 0.1 mmol) and a reaction time of 48 hours. The reaction mixture was analyzed directly by ^{19}F NMR (40 %, *o*/*m*/*p* = 1.4/1/3.1).
The typical reaction B was followed with benzonitrile (52 μL, 0.5 mmol), Na_2SO_4 (0.14 g, 1 mmol), 0.1wt% Rh/anatase TiO_2 (0.016 g, 0.2 mmol) and a reaction time of 48 hours. The reaction mixture was analyzed directly by ^{19}F NMR (11 %, *o*/*m*/*p* = 0/1/1.8).
Due to the availability of the products, no purification was attempted on this reaction mixture. The fluorine signals of the products were identical to those of the commercial samples.

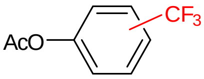

2m

The typical reaction A was followed with phenyl acetate (64μL, 0.5 mmol), $Na_2S_2O_8$ (0.0238 g, 0.1 mmol), 0.1wt% Rh/anatase TiO_2 (0.008 g, 0.1 mmol) and a reaction time of 48 hours. The reaction mixture was analyzed directly by ^{19}F NMR (47 %, *o*/*m*/*p* = 1/1.1/1.5).

The typical reaction B was followed with phenyl acetate (64μL, 0.5 mmol), Na_2SO_4 (0.14 g, 1 mmol), 0.1wt% Rh/anatase TiO_2 (0.016 g, 0.2 mmol) and a reaction time of 48 hours. The reaction mixture was analyzed directly by ^{19}F NMR (18 %, *o*/*m*/*p* = 1.4/1.2/1).

The identities of trifluoromethylated products of **2m** were verified by synthesis of authentic samples from their corresponding phenols in analogy to a literature procedure.[1]

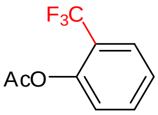

o-CF_3-**2m**

The product was purified as a colorless oil by distillation under vaccum. ^{1}H NMR (400 M, $CDCl_3$) δ 7.67 (d, J = 7.84 Hz, 1H), 7.58 (t, J = 7.76 Hz, 1H), 7.35 (t, J = 7.66 Hz, 1H), 7.24 (t, J = 8.70 Hz, 1H), 2.34 (s, 1H). ^{13}C NMR (101 M, $CDCl_3$) δ 168.88, 148.13(q, J_{C-F} = 2.00 Hz), 133.02, 126.91 (q, J_{C-F} = 4.81 Hz), 125.97, 124.45, 122.91(q, J_{C-F} = 31.47 Hz), 122.98(q, J_{C-F} = 272.60 Hz), 20.68. ^{19}F NMR (377 M, $CDCl_3$) δ -61.93 (s, 3F).

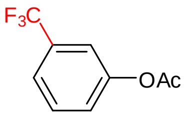

m-CF_3-**2m**

The product was purified as a colorless oil by distillation under vaccum. ^{1}H NMR (400 M, $CDCl_3$) δ 7.51-7.50 (m, 2H), 7.37 (s, 1H), 7.31-7.29 (m, 1H), 2.33 (s, 1H). ^{13}C NMR (101 M, $CDCl_3$) δ 169.19, 150.70, 131.97 (q, J_{C-F} = 32.99 Hz), 130.00, 125.22, 123.52 (q, J_{C-F} = 272.40 Hz), 122.68 (q, J_{C-F} = 3.66 Hz), 118.90 (q, J_{C-F} = 3.65 Hz), 21.00. ^{19}F NMR (377 M, $CDCl_3$) δ -62.72 (s, 3F).

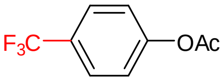

p-CF_3-**2m**

The product was purified as a colorless oil by distillation under vaccum. ^{1}H NMR (400 M, $CDCl_3$) δ 7.65 (d, J = 8.56 Hz, 2H), 7.22 (d, J = 8.48 Hz, 2H), 2.33 (s, 1H). ^{13}C NMR (101 M, $CDCl_3$) δ 168.92, 153.15, 128.10 (q, J_{C-F} = 32.82 Hz), 126.79 (q, J_{C-F} = 3.74 Hz), 123.87 (q, J_{C-F} = 272.01 Hz), 122.09, 21.05. ^{19}F NMR (377 M, $CDCl_3$) δ -62.27 (s, 3F).

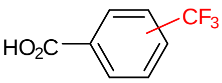

2n

The typical reaction A was followed with benzoic acid (0.061g, 0.5 mmol), $Na_2S_2O_8$ (0.0479 g, 0.2 mmol), 0.1wt% Rh/anatase TiO_2 (0.008 g, 0.1 mmol) and a reaction time of 48 hours. The reaction mixture was analyzed directly by ^{19}F NMR (34 %, *o*/*m*/*p* = 3.2/1/2.2).
The typical reaction B was followed with benzoic acid (0.061g, 0.5 mmol), Na_2SO_4 (0.14 g, 1 mmol), 0.1wt% Rh/anatase TiO_2 (0.008 g, 0.1 mmol) and a reaction time of 48 hours. The reaction mixture was analyzed directly by ^{19}F NMR (38 %, *o*/*m*/*p* = 3.2/1/1.2).
Due to the availability of the products, no purification was attempted on this reaction mixture. The fluorine signals of the products were identical to those of the commercial samples.

2o 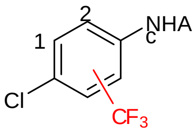

Typical reaction A was followed with 4'-chloroacetanilide (0.0848 g, 0.5 mmol), 0.1 wt% Rh/anatase TiO_2 (0.008 g, 0.1 mmol), $Na_2S_2O_8$ (0.0238 g, 0.1 mmol) and a reaction time of 48 hours to provide **2o** in 50 % yield (C2/C1 = 1/1).

Typical reaction B was followed with 4'-chloroacetanilide (0.0848 g, 0.5 mmol), 0.1 wt% Rh/anatase TiO_2 (0.016 g, 0.2 mmol), Na_2SO_4 (0.14 g, 1 mmol) and a reaction time of 48 hours to provide **2o** in 14 % yield (C2/C1 = 1.3/1).

The product was purified as a white solid by silica gel chromatography using eluant (50 % EtOAc/hexanes, **2o**-C1 R_f = 0.4, **2o**-C2 R_f = 0.56).

2o-C1 ^{1}H NMR (400 M, $CDCl_3$) 7.80 (s, 1H), 7.73 (d, J = 8.56 Hz, 1H), 7.58 (br s, 1H), 7.43 (d, J = 8.64 Hz, 1H), 2.20 (s, 3H). ^{13}C NMR (101 M, $CDCl_3$) δ 169.18, 136.85, 132.07, 128.78 (q, $J_{C\text{-}F}$ = 31.63 Hz), 127.02, 124.02, 122.64 (q, $J_{C\text{-}F}$ = 273.40 Hz), 118.99 (q, $J_{C\text{-}F}$ = 5.66 Hz), 24.53. ^{19}F NMR (377 M, $CDCl_3$) δ -62.85 (s, 3F).

2o-C2 ^{1}H NMR (400 M, $CDCl_3$) 8.17 (d, J = 8.12 Hz, 1H), 7.59 (d, J = 2.04 Hz, 1H), 7.52 (d, J = 8.68, 1H), 7.35 (br s, 1H), 2.22 (s, 3H). ^{13}C NMR (101 M, $CDCl_3$) δ 168.54, 133.92, 132.99, 130.12, 126.33 (q, $J_{C\text{-}F}$ = 5.60 Hz), 126.13, 123.31 (q, $J_{C\text{-}F}$ = 273.64 Hz), 121.64 (q, $J_{C\text{-}F}$ = 30.48 Hz), 27.76. ^{19}F NMR (377 M, $CDCl_3$) δ -61.03 (s, 3F).

2p 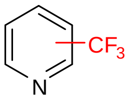

The typical reaction A was followed with pyridine (40 μL, 0.5 mmol), 0.1 wt% Rh/anatase TiO_2 (0.008 g, 0.1 mmol), $Na_2S_2O_8$ (0.0479 g, 0.2 mmol) and a reaction time of 48 hours. The reaction mixture was analyzed directly by ^{19}F NMR (55 %, *o*/*m*/*p* = 2.7/2.4/1).

The typical reaction B was followed with pyridine (40 μL, 0.5 mmol), 0.1 wt% Rh/anatase TiO_2 (0.016 g, 0.2 mmol), Na_2SO_4 (0.14 g, 1 mmol) and a reaction time of 48 hours. The reaction mixture was analyzed directly by ^{19}F NMR (50 %, *o*/*m*/*p* = 3.5/1/3.7).

Due to the availability of the products, no purification was attempted on this reaction mixture. The fluorine signals of the products were identical to those of the commercial samples.

2q 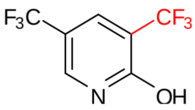

Typical reaction A was followed with 2-hydroxy-5-trifluoromethylpyridine (0.0816 g, 0.5 mmol), $Na_2S_2O_8$ (0.0479 g, 0.2 mmol), 0.1 wt% Rh/anatase TiO_2 (0.016 g, 0.2 mmol) and a reaction time of 48 hours to provide **2q** in 51 % yield.

Typical reaction B was followed with 2-hydroxy-5-trifluoromethylpyridine (0.0816 g, 0.5 mmol), Na_2SO_4 (0.14 g, 1 mmol), 0.1 wt% Rh/anatase TiO_2 (0.016 g, 0.2 mmol) and a reaction time of 48 hours to provide **2q** in 32 % yield.

The product was purified as a white solid by prep TLC using eluant (67 % EtOAc/hexanes, R_f = 0.6). ^{1}H NMR (400 M, $CDCl_3$) δ 8.02-8.03 (m, 2H). ^{13}C NMR (101 M, $CDCl_3$) δ 160.81, 138.23

(q, $J_{C\text{-}F}$ = 4.72 Hz), 137.02-136.94 (m), 122.47 (q, $J_{C\text{-}F}$ = 270.29 Hz), 121.68 (q, $J_{C\text{-}F}$ = 272.06 Hz), 121.39 (q, $J_{C\text{-}F}$ = 32.38 Hz), 110.14 (q, $J_{C\text{-}F}$ = 36.41 Hz). ^{19}F NMR (377 M, $CDCl_3$) δ -62.41 (s, 3F), -63.30 (s, 3F).

2r

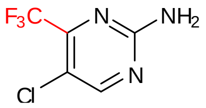

Typical reaction A was followed with 2-amino-5-chloropyrimidine (0.065 g, 0.5 mmol), 0.1 wt% Rh/anatase TiO_2 (0.016 g, 0.2 mmol), $Na_2S_2O_8$ (0.0238 g, 0.1 mmol), and a reaction time of 48 hours to provide **2r** in 37 % yield.
Typical reaction B was followed with 2-amino-5-chloropyrimidine (0.065 g, 0.5 mmol), 0.1 wt% Rh/anatase TiO_2 (0.016 g, 0.2 mmol), Na_2SO_4 (0.14 g, 1 mmol) and a reaction time of 48 hours to provide **2r** in 32 % yield.
The product was purified as a white solid by silica gel chromatography using eluant (33 % EtOAc/hexanes, R_f = 0.5). ^{1}H NMR (400 M, DMSO-D6) δ 8.59 (s, 1H), 7.55 (s, 2H).
^{13}C NMR (101 M, DMSO-D6) δ 162.00, 161.55, 150.52 (q, $J_{C\text{-}F}$ = 33.91 Hz), 120.60 (q, $J_{C\text{-}F}$ = 276.40 Hz), 113.08. ^{19}F NMR (377 M, $CDCl_3$) δ -68.40 (s, 3F).

2s

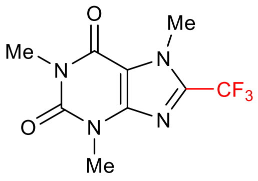

Typical gram scale reaction was followed to provide **2s** in 75 % yield.
Typical reaction B was followed with caffeine (0.0971 g, 0.5 mmol), 0.1 wt% Rh/anatase TiO_2 (0.016 g, 0.2 mmol), Na_2SO_4 (0.14 g, 1 mmol) and a reaction time of 48 hours to provide **2s** in 58 % yield.
The product was purified as a white solid by silica gel chromatography using eluant (EtOAc/hexanes = 1/1, R_f = 0.4). ^{1}H NMR (400 M, $CDCl_3$) δ 4.14 (d, J = 1.08 Hz, 3H), 3.57 (s, 3H), 3.40 (s, 3H). ^{13}C NMR (101 M, $CDCl_3$) δ 155.44, 151.32, 146.50, 138.90 (q, $J_{C\text{-}F}$ = 40.07 Hz), 118.73 (q, $J_{C\text{-}F}$ = 271.27 Hz), 109.63, 33.17 (d, $J_{C\text{-}F}$ = 1.60 Hz), 29.89, 28.18. ^{19}F NMR (377 M, $CDCl_3$) δ -62.42 (s, 3F).

2t

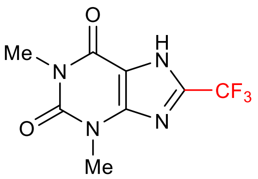

Typical gram scale reaction was followed with Theophylline (1.08 g, 6 mmol) to provide **2t** in 73 % yield.
Typical reaction B was followed with caffeine (0.09 g, 0.5 mmol), 0.1 wt% Rh/anatase TiO_2 (0.008 g, 0.2 mmol), Na_2SO_4 (0.14 g, 1 mmol) and a reaction time of 48 hours to provide **2t** in 48 % yield.
The product was purified as a white solid by silica gel chromatography using eluant

(EtOAc/hexanes = 2/1, R_f = 0.2). ^{1}H NMR (400 M, DMSO-D6) δ 3.43 (s, 3H), 3.25 (s, 3H). ^{13}C NMR (101 M, DMSO-D6) δ 155.10, 151.47, 147.30, 137.81 (q, J_{C-F} = 41.47 Hz), 118.64 (q, J_{C-F} = 269.96 Hz), 109.64, 30.37, 28.40. ^{19}F NMR (377 M, DMSO-D6) δ -62.66 (s, 3F).

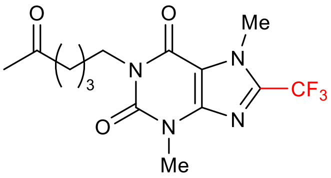

2u

Typical reaction A was followed with pentoxifylline (0.139 g, 0.5 mmol), 0.1 wt% Rh/anatase TiO_2 (0.016 g, 0.2 mmol), $Na_2S_2O_8$ (0.0479 g, 0.2 mmol) and a reaction time of 48 hours to provide **2u** in 67 % yield.

Typical reaction B was followed with pentoxifylline (0.139 g, 0.5 mmol), 0.1 wt% Rh/anatase TiO_2 (0.008 g, 0.1 mmol), Na_2SO_4 (0.14 g, 1 mmol) and a reaction time of 48 hours to provide **2u** in 63 % yield.

The product was purified as a white solid by silica gel chromatography using eluant (EtOAc/hexanes = 1/1, R_f = 0.35). ^{1}H NMR (400 M, $CDCl_3$) δ 4.03 (s, 3H), 3.86 (t, J = 6.6 Hz, 2H), 3.43 (s, 3H), 2.38 (t, J = 6.5 Hz, 2H), 2.01 (s, 3H), 1.51-1.50 (m, 4H).

^{13}C NMR (101 M, $CDCl_3$) δ 208.64, 155.10, 150.89, 146.38, 138.68 (q, J_{C-F} = 39.90 Hz), 118.07 (q, J_{C-F} = 271.20 Hz), 109.54, 42.83, 40.91, 33.01 (d, J_{C-F} = 1.29 Hz), 29.70, 29.60, 27.11, 20.69. ^{19}F NMR (377 M, $CDCl_3$) δ -62.43 (s, 3F).

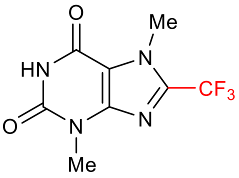

2v

Typical reaction was A followed with theobromine (0.09 g, 0.5 mmol), 0.1 wt% Rh/anatase TiO_2 (0.016 g, 0.2 mmol), $Na_2S_2O_8$ (0.0479 g, 0.2 mmol) and a reaction time of 48 hours to provide **2v** in 40 % yield.

Typical reaction was B followed with theobromine (0.09 g, 0.5 mmol), 0.1 wt% Rh/anatase TiO_2 (0.008 g, 0.1 mmol), Na_2SO_4 (0.14 g, 1 mmol) and a reaction time of 48 hours to provide **2v** in 45 % yield.

The product was purified as a white solid by silica gel chromatography using eluant (EtOAc/hexanes = 1/1, R_f = 0.25). ^{1}H NMR (400 M, $CDCl_3$) δ 9.08 (s, 1H), 4.14 (s, 3H), 3.55 (s, 3H). ^{13}C NMR (101 M, $CDCl_3$) δ 155.10, 150.93, 148.55, 139.63 (q, J_{C-F} = 40.25 Hz), 118.23 (q, J_{C-F} = 271.17 Hz), 110.16, 33.45(d, J_{C-F} = 2.15 Hz), 29.31. ^{19}F NMR (377 M, $CDCl_3$) δ -62.51 (s, 3F).

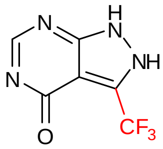

2w

Typical reaction A was followed with allopurinol (0.0681 g, 0.5 mmol), 0.1 wt% Rh/anatase TiO_2

(0.016 g, 0.2 mmol), $Na_2S_2O_8$ (0.0479 g, 0.2 mmol) and a reaction time of 48 hours to provide **2w** in 57 % yield.

Typical reaction B was followed with allopurinol (0.0681 g, 0.5 mmol), 0.1 wt% Rh/anatase TiO_2 (0.008 g, 0.1 mmol), 0.14 g Na_2SO_4 (1 mmol) and a reaction time of 48 hours to provide **2w** in 65 % yield.

The product was purified as a white solid by silica gel chromatography using eluant (EtOAc/hexanes = 2/1, R_f = 0.2). ^{1}H NMR (400 M, MeOD-D4) δ 8.07 (s, 1H). ^{13}C NMR (101 M, MeOD-D4) δ 156.59, 155.06, 148.66, 137.49 (q, $J_{C\text{-}F}$ = 40.12 Hz), 120.52 (q, $J_{C\text{-}F}$ = 268.49 Hz), 102.51. ^{19}F NMR (377 M, DMSO-D6) δ -60.89 (s, 3F).

Supplementary References

1. Cook, A. K., Emmert, M. H., & Sanford, M. S. Steric Control of Site Selectivity in the Pd-Catalyzed C–H Acetoxylation of Simple Arenes. *Org. Lett.* **15**, 5428-5431 (2013).
